# Bioinspired design of a polymer gel sensor for the realization of extracellular Ca^2+^ imaging

**DOI:** 10.1038/srep24275

**Published:** 2016-04-12

**Authors:** Fumitaka Ishiwari, Hanako Hasebe, Satoko Matsumura, Fatin Hajjaj, Noriko Horii-Hayashi, Mayumi Nishi, Takao Someya, Takanori Fukushima

**Affiliations:** 1 Chemical Resources Laboratory, Tokyo Institute of Technology, 4259 Nagatsuta, Midori-ku, Yokohama 226-8503, Japan; 2 Department of Anatomy and Cell Biology, Faculty of Medicine, Nara Medical University, Kashihara, Nara 634-8521, Japan; 3 Department of Electrical and Electronic Engineering, The University of Tokyo, 7-3-1 Hongo, Bunkyo-ku, Tokyo 113-8656, Japan

## Abstract

Although the role of extracellular Ca^2+^ draws increasing attention as a messenger in intercellular communications, there is currently no tool available for imaging Ca^2+^ dynamics in extracellular regions. Here we report the first solid-state fluorescent Ca^2+^ sensor that fulfills the essential requirements for realizing extracellular Ca^2+^ imaging. Inspired by natural extracellular Ca^2+^-sensing receptors, we designed a particular type of chemically-crosslinked polyacrylic acid gel, which can undergo single-chain aggregation in the presence of Ca^2+^. By attaching aggregation-induced emission luminogen to the polyacrylic acid as a pendant, the conformational state of the main chain at a given Ca^2+^ concentration is successfully translated into fluorescence property. The Ca^2+^ sensor has a millimolar-order apparent dissociation constant compatible with extracellular Ca^2+^ concentrations, and exhibits sufficient dynamic range and excellent selectivity in the presence of physiological concentrations of biologically relevant ions, thus enabling monitoring of submillimolar fluctuations of Ca^2+^ in flowing analytes containing millimolar Ca^2+^ concentrations.

Ca^2+^ plays a crucial role in many important physiological and pathological processes in animals^1^,^2^,^3^,^4^,^5^,^6^,^7^,^8^,^9^,^10^,^11^,^12^,^13^,^14^,^15^,^16^,^17^ and plants^9^,^18^,^19^,^20^,^21^,^22^,^23^. Over the past several decades, many synthetic molecular and genetically encoded fluorescent Ca^2+^ indicators have been developed, as represented by 1,2-bis(*o*-aminophenoxy)-ethane-*N*,*N*,*N*′,*N*′-tetraacetic acid (BAPTA) derivatives^24^,^25^,^26^,^27^ and calmodulin-based proteins^28^,^29^,^30^,^31^,^32^, respectively. Ca^2+^-imaging techniques that use such fluorescent indicators are indispensable in modern biology and medical science. In living organisms, Ca^2+^ concentrations differ greatly depending on the compartment. Typically, the Ca^2+^ concentration is ~100 nanomolar (nM) in intracellular cytosol, ~100 micromolar (*μ*M) in the endoplasmic reticulum and mitochondria and ~1 millimolar (mM) in extracellular fluid and blood (Fig. 1a,b)^3^. Plant vacuoles are also considered to contain mM-order Ca^2+^ concentrations^20^. Hence, Ca^2+^ imaging in all of these compartments requires dedicated fluorescent indicators with specific dissociation constants (*K*_d_) that are appropriate for the respective background Ca^2+^ concentrations. However, almost every Ca^2+^ indicator known to date has a *K*_d_ value ranging from nM to *μ*M, and therefore allows for Ca^2+^ imaging only in cytosol and organelles (Fig. 1a). Fluorescent Ca^2+^ indicators with mM-order *K*_d_, compatible with extracellular Ca^2+^ concentrations^27^,^32^, have scarcely been developed^9^,^10^, despite the fact that extracellular Ca^2+^, which is conventionally regarded as a diagnostic indicator for many diseases^3^,^7^, is now receiving considerable attention as a first messenger^3^,^4^,^5^,^6^,^7^,^8^,^9^,^10^,^11^,^12^,^13^,^14^,^15^,^16^,^17^ in, for example, parathyroid gland^3^,^4^, neuron^12^,^13^, myocyte^14^, stem cell^15^ and macrophages^16^,^17^.

In fact, there are major problems in the development of indicators for extracellular Ca^2+^ imaging^9^,^10^. First, such indicators should be designed to strike a balance between mM-order *K*_d_ (*i.e.*, a rather small affinity for Ca^2+^) and high selectivity for Ca^2+^ in the presence of excessive amounts of other physiological ions. Although simple Ca^2+^ imaging against mM-order background concentration of Ca^2+^ may be possible using existing indicators with *μ*M-order *K*_d_, Ca^2+^ indicators with one-order higher *K*_d_ have a great advantage in monitoring Ca^2+^ transients and oscillations in extracellular regions. Even more challenging in extracellular Ca^2+^ imaging, one has to create a mechanism to avoid the outflow of indicators from an observation area through molecular diffusion. Obviously, this issue is intractable with existing molecular-based indicators.

Here we report a conceptually new fluorescent Ca^2+^ sensor that can clear up all the above problems. It is a solid-state (gel) sensor that consists of a chemically-crosslinked polyacrylic acid (PAA), and its sensing mechanism relies not on conventional host-guest chemistry using tailored Ca^2+^-binding sites but on polymer-chain dynamics triggered by Ca^2+^. We show that ordinary PAA, when given pendants of tetraphenylethene (TPE), an aggregation-induced emission (AIE) luminogen^33^,^34^,^35^, becomes fluorescent in the presence of Ca^2+^. This series of polymers (PAA-TPE_*x*_, Fig. 1c) has mM-order apparent *K*_d_ for Ca^2+^ and can selectively sense Ca^2+^ against high background concentrations of physiological ions, glucose and amino acids. Remarkably, its chemically-crosslinked gel (g-PAA-TPE_*x*_, Fig. 1c,d) inherits the excellent Ca^2+^ selectivity and mM-order *K*_d_ of PAA-TPE_*x*_, thus providing a solid-state sensor that enables not only spatial imaging of Ca^2+^ in a macroscopic biological sample such as brain slices but also temporal detection of submillimolar fluctuations (±0.2 mM) in the Ca^2+^ concentration in a flowing analyte containing ~1 mM Ca^2+^.

## Results and Discussion

**Figure 1:**
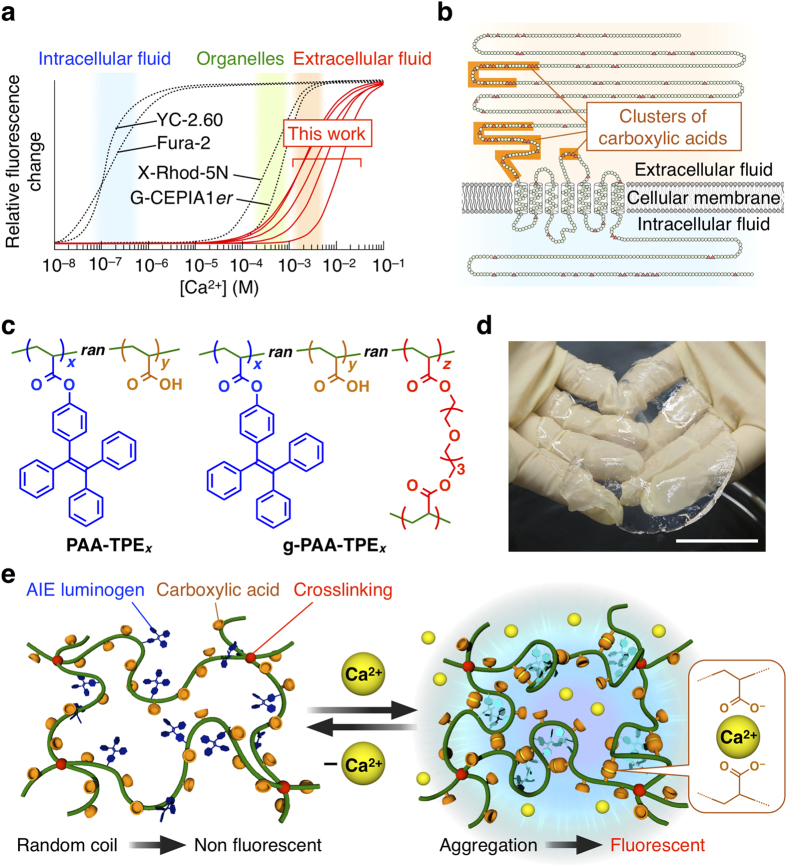
Design of Ca^2+^ sensors based on tetraphenylethene (TPE)-appended polyacrylic acid (PAA). (**a**) Schematic illustration showing the relationship between Ca^2+^ concentrations in biological systems and applicable concentration ranges of typical Ca^2+^ indicators (Fura-2^24^, X-Rhod-5N^25^, YC-2.60^29^ and G-CEPIA1*er*^31^). (**b**) Schematic illustration of the extracellular Ca^2+^-sensing receptor (CaSR)^4^. (**c**) Chemical structures of PAA-TPE_*x*_ and g-PAA-TPE_*x*_, where *x*, *y* and *z* indicate the molar ratios (contents) of TPE, PAA and crosslinker, respectively (see also Table 1), and *ran* means that the monomer sequence is random, *i.e.*, random copolymer. (**d**) Photograph of a sheet of swollen g-PAA-TPE_0.02_. Scale bar, 5 cm. (**e**) Schematic illustration of the mechanism of Ca^2+^ sensing with g-PAA-TPE_*x*_.

As a clue for the design of the new sensor, we took notice of CaSR, a natural extracellular Ca^2+^-sensing receptor^4^,^6^, which senses the change of Ca^2+^ concentration in extracellular regions and sends a signal to intracellular regions (Fig. 1b). Unlike calmodulin^36^,^37^, CaSR does not have particular high-affinity Ca^2+^-binding amino acid sequences^4^,^6^ and instead possesses highly acidic domains containing clusters of carboxylic acid functionalities (Fig. 1b). The acidic domains are believed to be responsible for Ca^2+^ binding. This holds true for the extracellular Ca^2+^-sensing receptor (CAS) in plants^21^ as well as other low-affinity Ca^2+^-binding proteins^38^,^39^. Regarding the interaction between Ca^2+^ and clustering carboxylic acid domains in CaSR and CAS, we found an interesting analogy with a work of Flory^40^, which had shown that the intrinsic viscosity of PAA in water decreases considerably upon addition of Ca^2+^ (25–50 mM). Subsequent reports, including those of Ikegami *et al.*^41^ and Huber *et al.*^42^,^43^, have indicated that mM-order Ca^2+^ causes the single-chain aggregation of PAA in water, for which [CO_2_^−^–Ca^2+^–^−^O_2_C]-type ion binding is responsible^44^. Importantly, among major ions in the body (Na^+^, K^+^, Mg^2+^ and Ca^2+^), Ca^2+^ most effectively triggers such conformational change of the PAA chain^40^,^41^,^42^,^43^.

Inspired by the analogy between natural and synthetic polymers, we designed PAA-TPE_*x*_ with an expectation that the conformational change of PAA chain between aggregation and expansion upon binding and release of Ca^2+^, respectively, might be translated into the fluorescence property of the TPE pendants (Fig. 1e). AIE luminogens, in contrast to usual fluorescent dyes, are known to fluoresce upon aggregation and are only weakly fluorescent in the molecularly dispersed state^33^,^34^,^35^. We also conceived that, if such a polymer-based indicator could be properly crosslinked, the resultant gel (a macroscopic material) might serve as a solid-state Ca^2+^ sensor with mM-order *K*_d_, which allows for long-term monitoring of extracellular Ca^2+^ dynamics at the organ level.

**Figure 2:**
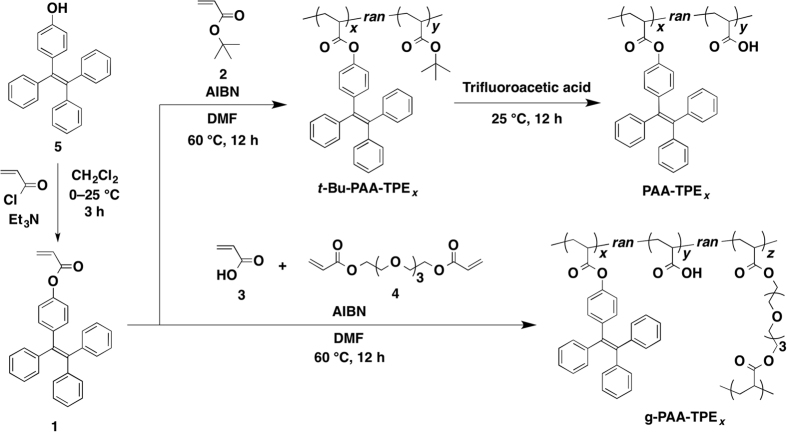
Synthetic scheme of PAA-TPE_*x*_ and g-PAA-TPE_*x*_.

Random copolymers PAA-TPE_*x*_ (Fig. 1c, Table 1, entries 1–5) containing 1–5 mol% (*x* = 0.01–0.05) of TPE pendants were synthesized by a two-step procedure involving the free-radical copolymerization of TPE-appended acrylate **1** and *t*-butyl acrylate **2** with the corresponding feed ratio (**1**/**2** = 1/99–5/95) and the subsequent removal of *t*-butyl groups from the resulting copolymers using trifluoroacetic acid (Fig. 2, see Methods for details). The chemical structure of PAA-TPE_*x*_ was unambiguously characterized by nuclear magnetic resonance (NMR) and infrared (IR) spectroscopy (Supplementary Figs S20 and S21). By means of gel permeation chromatography (GPC) using polystyrene standards, we estimated the number mean molecular weight (*M*_n_) of PAA-TPE_*x*_ to be approximately 20 kDa (Table 1, entries 1–5).

**Figure 3:**
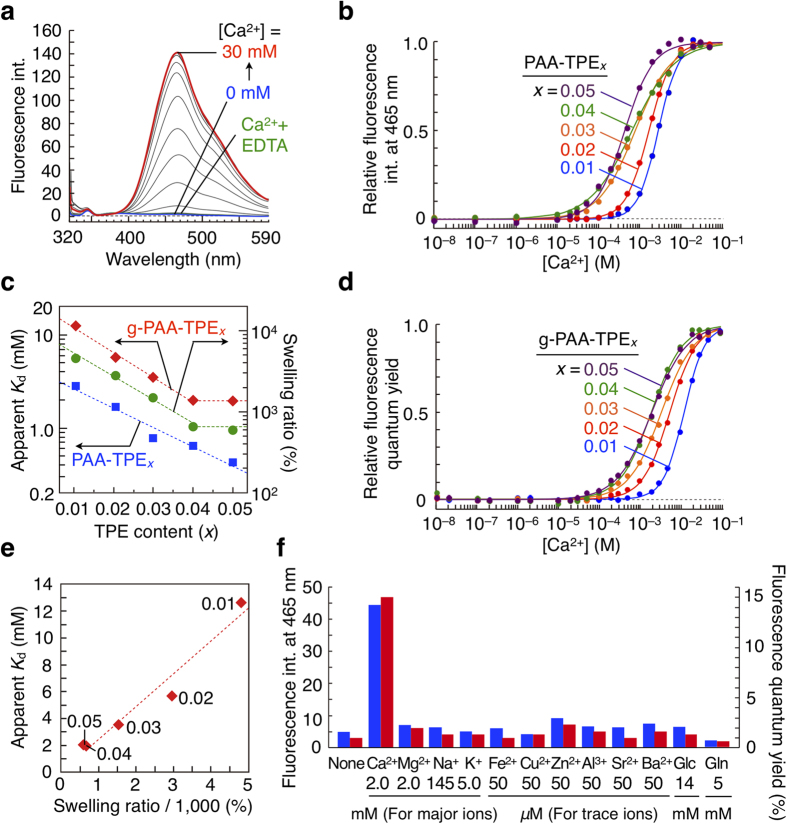
Ca^2+^-sensing properties of PAA-TPE_*x*_ and g-PAA-TPE_*x*_. (**a**) Fluorescence spectral changes of PAA-TPE_0.02_ (10 mg/L) in a HEPES buffer solution (70 mM, pH = 7.4) at 25 °C upon addition of CaCl_2_ (blue: 0 mM → red: 30 mM), and after further addition of EDTA (green: 30 mM). The wavelength of absorption maximum (307 nm) due to the TPE chromophore is essentially unchanged upon addition of CaCl_2_ (Supplementary Fig. S1). (**b**) Ca^2+^ titration curves of PAA-TPE_*x*_ (10 mg/L) in a HEPES buffer solution (70 mM, pH = 7.4). The relative fluorescence intensity is defined as (*F*–*F*_min_)/(*F*_max_–*F*_min_), where *F*, *F*_max_ and *F*_min_ represent observed, maximum and minimum fluorescence intensities, respectively. (**c**) Plots of the logarithms of the apparent *K*_d_ values of PAA-TPE_*x*_ (blue) and g-PAA-TPE_*x*_ (red) versus TPE contents, and plots of the logarithms of the swelling ratios of g-PAA-TPE_*x*_ (green) versus TPE contents. (**d**) Ca^2+^ titration curves of g-PAA-TPE_*x*_ (5 mg) in a HEPES buffer solution (70 mM, 5 mL, pH = 7.4). (**e**) Plot of apparent *K*_d_ of g-PAA-TPE_*x*_ versus the swelling ratio. (**f**) Fluorescence intensities of PAA-TPE_0.02_ (blue bars) and fluorescence quantum yields of g-PAA-TPE_0.02_ (red bars) in the presence of various metal chlorides, glucose (Glc, 14 mM) and glutamine (Gln, 5 mM). [CaCl_2_] = [MgCl_2_] = 2 mM, [NaCl] = 145 mM, [KCl] = 5 mM, [FeCl_2_] = [CuCl_2_] = [ZnCl_2_] = [AlCl_3_] = [SrCl_2_] = [BaCl_2_] = 50 *μ*M.

**Figure 4:**
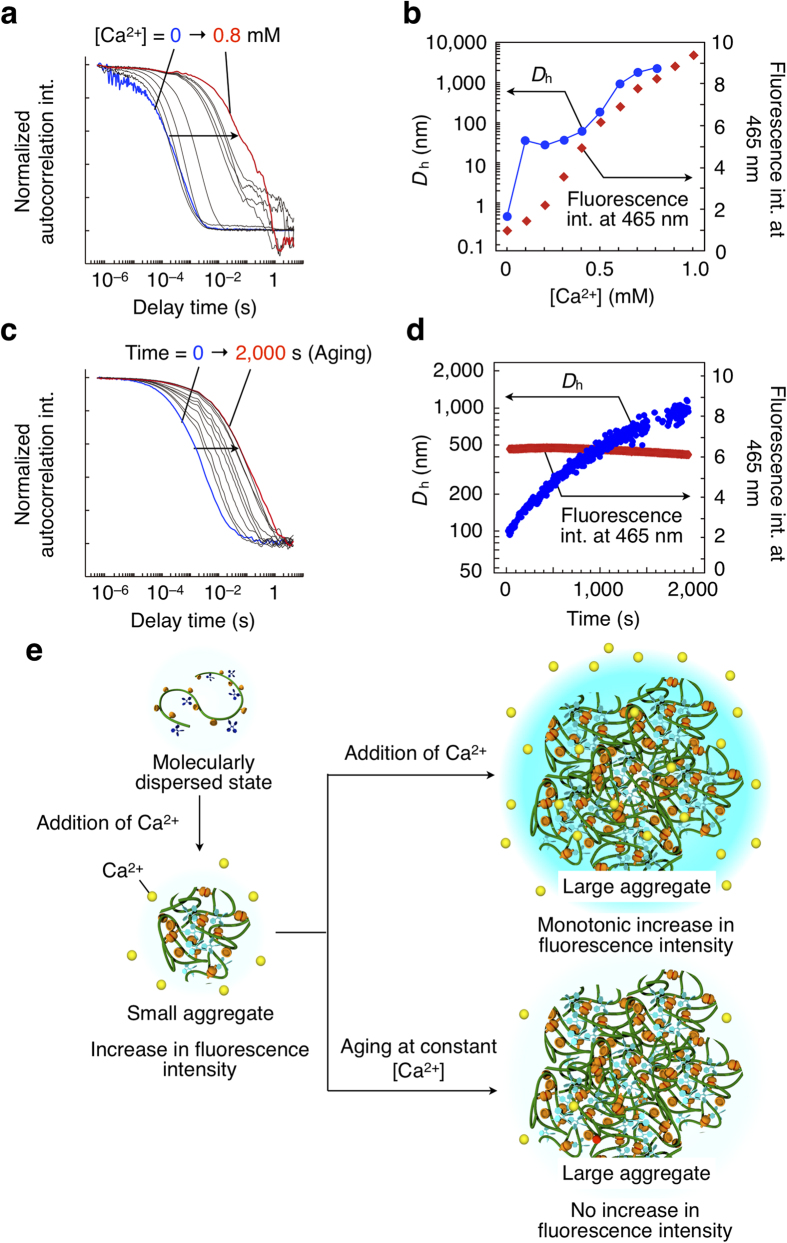
Aggregation behavior of PAA-TPE_0.02_ in the presence of Ca^2+^. (**a**) Changes in the autocorrelation functions of PAA-TPE_0.02_ (10 mg/L) at 25 °C in a water/methanol mixture (1/1 v/v) containing various concentrations of CaCl_2_ obtained by dynamic light scattering (DLS) measurements. (**b**) Ca^2+^-concentration dependence of the logarithms of hydrodynamic diameter (*D*_h_) and fluorescence intensity of PAA-TPE_0.02_ at 465 nm. (**c**) Time-dependent changes in the autocorrelation functions at 25 °C of PAA-TPE_0.02_ (10 mg/L) in a water/methanol mixture (1/1 v/v) containing 0.4 mM of CaCl_2_. (**d**) Time dependence of the logarithms of hydrodynamic diameter (*D*_h_) and fluorescence intensity of PAA-TPE_0.02_ at 465 nm. (**e**) Schematic illustration of a plausible aggregation behavior of PAA-TPE_0.02_ in the presence of Ca^2+^.

Although PAA-TPE_*x*_ (10 mg/L) in a buffer solution ([4-(2-hydroxyethyl)-1-piperazineethanesulfonic acid (HEPES)] = 70 mM, pH = 7.4) scarcely fluoresces, it becomes fluorescent upon addition of CaCl_2_. For example, the fluorescence intensity of PAA-TPE_0.02_ increased monotonically as the Ca^2+^ concentration was increased from 0.01 to 10 mM (Fig. 3a). As shown in the Ca^2+^ titration curves (Fig. 3b), the increase in fluorescence intensity occurred regardless of the TPE content (*x* = 0.01–0.05) (Table 1, entries 1–5). When PAA-TPE_*x*_ loses Ca^2+^, its polymer chain returns to a weakly fluorescent random-coil state. As soon as ethylenediaminetetraacetate (EDTA), a strong chelator for Ca^2+^ (*K*_d_ = *ca*. 10^–10 ^M), was added to a buffer solution containing, *e.g.*, PAA-TPE_0.02_ (10 mg/L) and Ca^2+^ (30 mM), the fluorescence was mostly quenched (Fig. 3a, green line). All of the above observations demonstrate that the Ca^2+^-triggered aggregation of the PAA chain is reflected in the fluorescence intensity of TPE. Notably, even PAA-TPE_0.01_, which has a TPE content of only 1 mol%, can successfully visualize the change in Ca^2+^ concentration. Dynamic light scattering (DLS) experiments confirmed that the increase in the fluorescence intensity of PAA-TPE_0.02_ is due to single-chain aggregation^40^,^41^,^42^,^43^ rather than interpolymer aggregation. As shown in Fig. 4a,b, when Ca^2+^ concentration was increased, the fluorescence intensity as well as the particle size (hydrodynamic diameter, *D*_h_) of PAA-TPE_0.02_ increased (Fig. 4b,e). In contrast, on aging at 25 °C with a constant Ca^2+^ concentration (*e.g.*, 0.4 mM), the particle size of PAA-TPE_0.02_ increased (Fig. 4c,d, blue symbols), while its fluorescence intensity remained almost unchanged (Fig. 4d, red symbols and Fig. 4e).

Fitting the Ca^2+^ titration curve (Fig. 3b and Supplementary Fig. S2a) using Hill’s equation provided the apparent *K*_d_ of PAA-TPE_*x*_ for Ca^2+^ (see Methods for details). As shown in Table 1 (entries 1–5), the values were all on the order of mM and ranged from 0.43 to 2.8 mM depending on the TPE content (*x*). The apparent *K*_d_ decreased as the TPE content increased, suggesting that the hydrophobic nature of TPE promotes the Ca^2+^-triggered single-chain aggregation of PAA-TPE_*x*_. Importantly, because the relationship between the logarithm of the apparent *K*_d_ and the TPE content is approximately linear (Fig. 3c, blue symbols), the apparent *K*_d_ of PAA-TPE_*x*_ can be continuously tuned in the range between 0.43 and 2.8 mM by simply varying the TPE content (*x*). This feature is beneficial for detecting a change in the Ca^2+^ concentration against various background concentrations of ions and provides an interesting contrast to typical molecular indicators such as the Fura series^25^, the *K*_d_ values of which are controlled by the electronic properties of the substituents on the BAPTA skeleton.

The single-chain aggregation of PAA-TPE_*x*_ and in turn the enhancement of fluorescence intensity occurs very selectively for Ca^2+^. Without Ca^2+^, PAA-TPE_*x*_ is weakly fluorescent in the presence of high concentrations of major ions in the body, *i.e.*, Na^+^ (145 mM), K^+^ (5 mM) and Mg^2+^ (2 mM), as well as a physiological concentration (50 *μ*M) of trace ions, *i.e.*, Fe^2+^, Cu^2+^, Zn^2+^, Al^3+^, Sr^2+^ and Ba^2+^ (Fig. 3f and Supplementary Fig. S3a,b). Moreover, glucose (14 mM) and all the natural amino acids (5 mM) did not significantly influence the fluorescence property of PAA-TPE_*x*_ (Fig. 3f and Supplementary Fig. S3a–d). To further test the selective sensing capability of PAA-TPE_*x*_, we performed a Ca^2+^ titration experiment using a buffer solution ([HEPES] = 70 mM, pH = 7.4) containing PAA-TPE_0.02_ (10 mg/L), Na^+^ (145 mM), K^+^ (5 mM), Mg^2+^ (2 mM), glucose (14 mM) and glutamine (5 mM). Upon addition of CaCl_2_, the fluorescence of the buffer solution of PAA-TPE_0.02_ intensified (Supplementary Fig. S4a). Note that PAA-TPE_*x*_ can recognize Ca^2+^ selectively in the presence of such a high concentration of Mg^2+^ (Supplementary Figs S5 and S6). Based on the titration curve (Supplementary Fig. S4b), the apparent *K*_d_ and the dynamic range (ratio of the maximum to the minimum fluorescence intensity) were determined to be 9.2 mM and 25, respectively. The difference of the apparent *K*_d_ for Ca^2+^ in the presence (9.2 mM) and absence (1.8 mM) of the biologically relevant ions and sugar likely arises from competing interactions of the carboxyl group of PAA with other metal ions and/or the polar functionalities of glucose and amino acid. This trend is generally observed for existing Ca^2+^ indicators^25^. From Ca^2+^ titration experiments under different pH (*e.g.*, pH = 7.0 and 8.1) and temperature (25–40 °C) conditions, we confirmed that such pH and temperature changes exert little influence on the Ca^2+^ sensing property of PAA-TPE_0.02_ (Supplementary Figs S7 and S8).

The mM-order *K*_d_, excellent selectivity and sufficient dynamic range of PAA-TPE_*x*_ for Ca^2+^ fulfill the essential requirements of sensing Ca^2+^ against high background concentrations of physiological ions. For the subsequent challenge in realizing a solid-state Ca^2+^ sensor, we prepared a chemically-crosslinked gel of PAA-TPE_*x*_. Typically, TPE-appended acrylate **1** (2 mol%) and acrylic acid **3** (95 mol%) were copolymerized in the presence of tetraethyleneglycol diacrylate **4** (3 mol%) as a crosslinker (Fig. 2, see Methods for details). The resultant gel (g-PAA-TPE_0.02_) was insoluble but swelled in aqueous media and could be readily processed into various shapes and sizes such as large-area flexible sheets and micro particles (Supplementary Fig. S9). When a droplet of a buffer solution of CaCl_2_ (30 mM) was placed on a large-area gel sheet, blue fluorescence emerged (Supplementary Movie S1), clearly demonstrating that PAA-TPE_0.02_, even when chemically crosslinked, can respond to Ca^2+^. The Ca^2+^-sensing property of g-PAA-TPE_0.02_ was largely dependent on the total monomer concentration in the copolymerization rather than the feed ratio of the crosslinker. We optimized the preparation conditions in terms of the total monomer concentration as well as the feed ratio of the crosslinker (Supplementary Fig. S10) so that the dynamic range could be maximized (Supplementary Fig. S11). The best results (dynamic range = 12) were obtained when the total monomer concentration was 1.5 M and the feed ratio of the crosslinker was 3 mol% (Supplementary Fig. S11a, entry 9 and Supplementary Fig. S11b, red block). We found that g-PAA-TPE_0.02_ thus obtained was the most swollen (Supplementary Fig. S11c, red symbol). Conversely, copolymerization at high total monomer concentrations (*e.g.*, 4.0 M) resulted in a less-swollen gel that scarcely responded to Ca^2+^ (Supplementary Fig. S11a, entries 1–4, Supplementary Fig. S11b, blue blocks and Supplementary Fig. S11c, blue symbols). Because the degree of polymer-chain entanglement generally decreases when the total monomer concentration is decreased^45^, g-PAA-TPE_0.02_, obtained under the optimized conditions (Supplementary Fig. S11a, entry 9), may maintain the mobility of the polymer chains to a large extent and undergo conformational changes upon binding to Ca^2+^. Meanwhile, the change in the feed ratio of the crosslinker did not impact largely on the dynamic range and swelling ratio of g-PAA-TPE_0.02_ (Supplementary Fig. S11a, *e.g.*, entries 1–4), indicating that the physical crosslinking due to polymer-chain entanglement is more important than the chemical crosslinking in determining the mobility of the polymer chain^45^ and in turn Ca^2+^-sensing properties of the gel.

**Figure 5:**
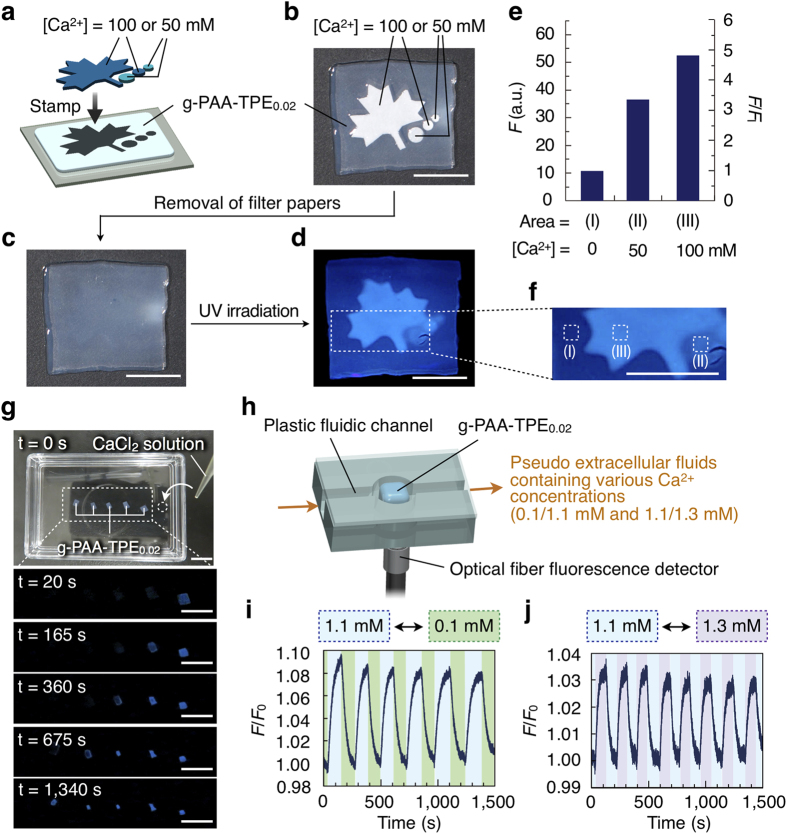
Spatiotemporal Ca^2+^-sensing capability of g-PAA-TPE_*x*_. **(a**) Schematic illustration of the stamp experiment using filter papers impregnated with CaCl_2_ aqueous solution: filter papers impregnated with either 50 or 100 mM CaCl_2_ solution were put on a gel sheet of g-PAA-TPE_0.02_. (**b–d,f**) Pictures of each experimental step: attachment of the gel to the filter papers (**b**), the gel sheet after removal of the papers (**c**), a fluorescent image under UV irradiation (**d**) and its magnification (**f**). Scale bars, 1.0 cm. (**e**) Fluorescence intensities (*F*; average brightness per area) and increasing ratio (*F*/*F*_I_) of three different areas of g-PAA-TPE_0.02_: (I) filter paper-non-attached area (background), (II) 50 mM CaCl_2_-attached area and (III) 100 mM CaCl_2_-attached area shown in (**f**) *F*_I_ represents fluorescence intensity of area (I). Scale bars, 1.0 cm. (**g**) Real-time fluorescence Ca^2+^ imaging with g-PAA-TPE_0.02_. Five gel sheets of g-PAA-TPE_0.02_ were immobilized on a Petri dish. An aqueous solution of CaCl_2_ (100 mM, 200 *μ*M) was dropped at the right side of the rightmost gel and the time course of changes in the fluorescence of the gel sheets was monitored. Scale bars, 1.0 cm. (**h**) Schematic illustration of the experimental setup for continuous monitoring of changes in the Ca^2+^ concentration using g-PAA-TPE_0.02_. (**i**,**j**) Temporal changes in the fluorescence intensity of g-PAA-TPE_0.02_ in response to alternating changes in the Ca^2+^ concentration (1.1/0.1 mM and 1.1/1.3 mM for **i** and **j**, respectively) in a flowing pseudo artificial extracellular fluid (6.7 mL/min) containing Na^+^ (145 mM), K^+^ (5 mM), Mg^2+^ (2 mM) and glucose (14 mM). *F* and *F*_0_ represent observed and initial fluorescence intensities, respectively. The small fluorescence decay of g-PAA-TPE_0.02_ upon prolonged UV irradiation is likely due to a photoreaction of the TPE units^48^.

At the optimal total monomer concentration (1.5 M), **1** and acrylic acid **3** were copolymerized with varying molar ratios (**1**/**3** = 1/96–5/92) in the presence of tetraethyleneglycol diacrylate **4** (3 mol%). The resultant materials (g-PAA-TPE_*x*_, *x* = 0.01–0.05) were all swollen in aqueous media and capable of sensing Ca^2+^ selectively (Fig. 3f). Table 1 (entries 6–10) summarizes the apparent *K*_d_ and the dynamic range of g-PAA-TPE_*x*_ as determined by titration experiments (Fig. 3d and Supplementary Fig. S2b). Importantly, each g-PAA-TPE_*x*_ has an apparent *K*_d_ value comparable to that of the corresponding non-crosslinked PAA-TPE_*x*_. As in the case of PAA-TPE_*x*_, the logarithms of the apparent *K*_d_ values correlated linearly with the TPE contents of g-PAA-TPE_*x*_ (*x* < 0.05, Fig. 3c, red symbols), indicating that the sensitivity of the gel sensor is tunable. The swelling ratios also correlated linearly with the TPE contents of g-PAA-TPE_*x*_ (*x* < 0.05, Fig. 3c, green symbols). Consequently, the apparent *K*_d_ values were proportional to the swelling ratios (Fig. 3e). The TPE-content dependence of the apparent *K*_d_ and swelling ratio most likely originates from the hydrophobic nature of TPE. We presume that the hydrophobic TPE pendants pre-aggregate in the swollen gel even without Ca^2+^ to engage in crosslinking of the polymer chains non-covalently (*i*.*e*., pseudo crosslinking). Hence, high-level loading of the TPE pendant (*x* = 0.05) would result in a decrease in the degree of freedom of the polymer chains to decrease the dynamic range. In fact, less-swollen g-PAA-TPE_0.05_ exhibited the smallest dynamic range among g-PAA-TPE_*x*_ (Table 1, entry 10).

g-PAA-TPE_*x*_ could be used in various sizes and shapes (Supplementary Fig. S9). For example, a gel sheet fabricated from g-PAA-TPE_0.02_ allowed spatial visualization of the Ca^2+^-concentration distribution. A simple stamp experiment, using shaped filter papers impregnated with two aqueous solutions with different Ca^2+^ concentrations (Fig. 5a–d), demonstrated that the difference in the Ca^2+^ concentration can be distinguished with the naked eye as a difference in fluorescence intensity (Fig. 5d–f). A stamp experiment using biological samples may demonstrate the potential of the gel sensor in biomedical applications. In this context, we observed subtle fluorescence behavior of g-PAA-TPE_0.02_ in a titration experiment using an albumin protein (bovine serum albumin, BSA). At BSA concentrations below 1.0 g/L, the fluorescence intensity of the gel monotonically increased, and then gradually decreased, mostly recovering its initial value at a BSA concentration of 20 g/L (Supplementary Fig. S12a). At this stage, upon subsequent addition of Ca^2+^, the gel turned fluorescent again (Supplementary Fig. S12b). Although the origin of these observations is unclear, we found that the influence of the protein on the Ca^2+^-sensing property of the gel can be avoided by covering the gel surface with a dialysis membrane, which may prevent proteins from contacting the gel. For instance, when a mouse brain slice was put on a gel sheet of g-PAA-TPE_0.02_ through a dialysis membrane and then removed, brain-shaped fluorescence emerged on the gel sheet (Supplementary Fig. S13a,b,d). As a control experiment, when a mouse brain slice was immersed in an EDTA solution for removing Ca^2+^ and then likewise stamped on a gel sheet of g-PAA-TPE_0.02_, minimal fluorescence was observed from the gel (Supplementary Fig. S13c,d). *In situ* imaging with fluorescence microscopy successfully visualized the microscopic distribution of Ca^2+^ in the brain slice (Supplementary Fig. S14).

Using gel sheets immobilized on a vessel, the diffusion of Ca^2+^ in a buffer solution could be visualized spatiotemporally (Fig. 5g and Supplementary Movie S2). To further examine the feasibility of the stationary detection of a change in the Ca^2+^ concentration in a flowing analyte, we monitored fluorescence in a microtomed section of g-PAA-TPA_0.02_ immobilized in a microfluidic channel (Fig. 5h and Supplementary Figs S15 and S16). Traumatic events such as epileptic seizures and terminal anoxia are accompanied by 1 mM-level changes in the Ca^2+^ concentration in the extracellular fluid inside the brain^46^. For a model experiment, we prepared, as a pseudo extracellular fluid, two buffer solutions of a mixture of physiological ions ([Na^+^] = 145 mM, [K^+^] = 5 mM, [Mg^2+^] = 2 mM and [glucose] = 14 mM) containing 1.1 or 0.1 mM Ca^2+^. When these solutions were alternately flowed through the microfluidic channel with the gel, reversible changes in fluorescence intensity were observed in response to changes in the Ca^2+^ concentration (Fig. 5i). More surprisingly, the gel recognized a small fluctuation (±0.2 mM) of the Ca^2+^ concentration against high background concentrations of physiological ions. Figure 5j shows serially repeated changes in fluorescence intensity under the alternating flow of pseudo extracellular fluids containing 1.1 or 1.3 mM Ca^2+^. Such a submillimolar fluctuation in the Ca^2+^ concentration is known to be associated with normal brain activity^47^. This result demonstrates the great potential of g-PAA-TPE_*x*_ as a tool for realizing extracellular Ca^2+^ imaging.

## Conclusion

We have demonstrated that conventional polyacrylic acid (PAA), when an aggregation-induced emission luminogen is attached to its main chain, provides a state-of-the-art solid-state fluorescent Ca^2+^ sensor, which can selectively detect submillimolar fluctuations of Ca^2+^ concentration. The fact that acidic domains with clustering carboxylic acid groups exist ubiquitously in natural extracellular Ca^2+^-sensing receptors as well as low-affinity Ca^2+^-binding proteins inspired us to use ordinary PAA that has been long known to undergo single-chain aggregation in the presence of mM-order Ca^2+^. The gel sensor is easy to synthesize at a large scale (Fig. 1d), has high processability (Supplementary Fig. S9), and can exert its superb function at high Ca^2+^ concentration even in the presence of competing amounts of alkali, alkaline-earth metal ions, sugars and amino acids. Considering its high potential, the gel sensor may serve as the first imaging tool for investigating the hitherto unexplored field of fluorescence extracellular Ca^2+^ imaging, eventually leading to comprehensive understanding of biological events involving Ca^2+^, particularly at the macroscopic organ levels. Besides the biological applications, the present sensor may be used in more general fields such as food and environmental inspection^25^,^49^.

## Methods

### Materials

Unless otherwise noted, all the commercial reagents were used as received. Prior to use, *t*-butyl acrylate (**2**), acrylic acid (**3**), tetraethylene glycol diacrylate (**4**), 1,4-butanediol diacrylate (**7**) and 1,10-decanediol diacrylate (**8**) were purified by passage through Al_2_O_3_ column to remove polymerization inhibitors. Azobisisobutyronitrile (AIBN) and *N*,*N*′-methylenebis-acrylamide (**6**) were purified by recrystallization from methanol. 4-(1,2,2-triphenylvinyl)phenol (**5**) was prepared according to the reported procedure^50^.

### Synthesis

(See Fig. 2).

#### 4-(1,2,2-triphenylvinyl)phenyl acrylate (1)

A CHCl_3_ (5 mL) solution of acryloyl chloride (0.42 mM, 5.2 mmol) was added dropwise at 0 °C to a CHCl_3_ (30 mL) solution of a mixture of 4-(1,2,2-triphenylvinyl)phenol (**5**, 910 mg, 35 mmol) and triethylamine (Et_3_N, 1.46 mL, 10 mmol). The resulting mixture was stirred at 25 °C for 3 h, poured into a saturated aqueous solution of NaHCO_3_, and then extracted with CHCl_3_. A combined organic extract was washed with water and brine, dried over anhydrous MgSO_4_, and then evaporated to dryness under a reduced pressure. The residue was subjected to column chromatography (SiO_2_, hexane/CHCl_3_ 1/1 v/v) to allow the isolation of **1** as white solid (756 mg) in 72% yield: ^1^H NMR (400 MHz, CDCl_3_) *δ* (ppm) 7.01–7.11 (m, 15H), 6.89 (d, *J* = 9.0 Hz, 2H), 6.56 (dd, *J* = 17.3, 1.3 Hz, 1H), 6.27 (dd, *J* = 10.5, 17.3 Hz, 1H), 5.99 (dd, *J* = 10.5, 1.3 Hz, 1H). ^13^C NMR (100 MHz, CDCl_3_) *δ* (ppm) 164.3, 149.0, 143.7, 143.6, 143.5, 141.4, 141.3, 140.0, 132.4, 132.3, 131.4, 131.3, 128.1, 127.9, 127.8, 127.7, 126.6, 126.5, 120.7. FT-IR (KBr) ν (cm^−1^) 3076, 3054, 3024, 1756, 1677, 1599, 1502, 1443, 1356, 1200, 1166, 1140, 1017, 763, 748, 699, 613, 572, 498. HRMS (FAB): calcd. for C_29_H_22_O_2_ [M]^+^ *m*/*z* = 402.1620; found: *m*/*z* = 402.1617.

#### *t*-Bu-PAA-TPE_*x*_

Typically, a dimethylformamide (DMF) solution (1.53 mL) of a mixture of monomer **1** (21.3 mg, 53 *μ*mol), *t*-butyl acrylate (**2**, 146 *μ*L, 1.0 mmol) and AIBN (1.7 mg, 11 *μ*mol) was degassed by freeze-pump-thaw cycles (three times) and purged with argon. The mixture was stirred at 60 °C for 12 h, allowed to cool to 25 °C, and then evaporated to dryness under a reduced pressure. The residue was freeze-dried from toluene to afford *t*-Bu-PAA-TPE_0.05_ quantitatively as white solid (167 mg): ^1^H NMR (400 MHz, CDCl_3_) *δ* (ppm) 6.79–7.11 (br), 2.05–2.39 (br), 1.71–1.86 (br), 1.20–1.63 (br). FT-IR (KBr) ν (cm^–1^) 2979, 2935, 1731, 1481, 1457, 1393, 1368, 1257, 1149, 1034, 909, 846, 751, 701, 471, 430. Using a procedure similar to that for *t*-Bu-PAA-TPE_0.05_, *t*-Bu-PAA-TPE_0.01–0.04_ were obtained quantitatively from monomer **1**, *t*-butyl acrylate (**2**) and AIBN with the corresponding monomer feed ratios. The values of *M*_n_ and PDI of *t*-Bu-PAA-TPE_*x*_, evaluated by GPC analysis, are summarized in Table 1.

#### PAA-TPE_*x*_

Typically, a trifluoroacetic acid (58 *μ*L) was added to *t*-Bu-PAA-TPE_0.05_ (10.0 mg, 78 *μ*mol). The mixture was stirred at 25 °C for 12 h and evaporated to dryness under a reduced pressure. The residual volatile compounds were azeotropically removed with methanol (100 mL, five times) to afford PAA-TPE_0.05 _quantitatively as white solid (9.8 mg): ^1^H NMR (400 MHz, CD_3_OD) *δ* (ppm) 6.79–7.21 (br), 2.28–2.65 (br), 1.40–2.22 (br). FT-IR (KBr) ν (cm^−1^) 2961, 2361, 1716, 1503, 1454, 1417, 1249, 1168, 802, 701, 614, 503, 414. Using a procedure similar to that for PAA-TPE_0.05_, PAA-TPE_0.01–0.04_ were obtained quantitatively from trifluoroacetic acid and the corresponding precursors (*t*-Bu-PAA-TPE_0.01–0.04_). The composition ratios of PAA-TPE_0.01–0.04_, evaluated by ^1^H NMR spectroscopy, are summarized in Table 1.

#### g-PAA-TPE_*x*_

Typically, a DMF (0.71 mL) solution of a mixture of monomer **1** (21.3 mg, 53 *μ*mol), acrylic acid (**3**, 67 *μ*L, 980 *μ*mol), tetraethylene glycol diacrylate (**4**, 8.6 *μ*L, 32 *μ*mol) and AIBN (1.7 mg, 11 *μ*mol) was degassed by freeze-pump-thaw cycles (three times) and purged with argon. The mixture was allowed to stand at 60 °C for 12 h and then cool to 25 °C. The resultant gelatinous material was subjected to Soxhlet extraction with a mixture of methanol/acetone (1/1 v/v) for 24 h, dried at 80 °C under a reduced pressure for 48 h, affording g-PAA-TPE_0.05_ as white solid (46 mg) in 48% yield. Using a procedure similar to that for g-PAA-TPE_0.05_, g-PAA-TPE_0.01–0.04_ were obtained in ~50% yield from monomer **1**, acrylic acid (**3**), tetraethylene glycol diacrylate (**4**) and AIBN with the corresponding monomer feed ratios. The feed ratios for the preparation of g-PAA-TPE_*x*_ are summarized in Table 1. Using procedures similar to that for g-PAA-TPE_*x*_, other crosslinked polymers (Supplementary Fig. S10) were obtained in ~50% yield from monomer **1**, acrylic acid (**3**), corresponding crosslinker (**6**–**8**) and AIBN with the corresponding monomer feed ratios.

### Evaluation of the apparent dissociation constant (*K*_d_)

Ca^2+^ titration curves were obtained by measuring the fluorescence intensities (for PAA-TPE_*x*_) or quantum yields (for g-PAA-TPE_*x*_) under various Ca^2+^ concentrations. Because the number of effective Ca^2+^-binding sites in PAA-TPE_*x*_ and g-PAA-TPE_*x*_ cannot be determined, a general stoichiometric analysis for determining the dissociation constant (*K*_d_) is not applicable to these systems. Instead, we used the apparent *K*_d_, which was obtained by fitting the Ca^2+^ titration curves with the following Hill’s equation (1) using the least square method in R software (http://www.R-project.org/).


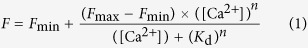


*F*: Fluorescence intensity (for PAA-TPE_*x*_) or quantum yield (for g-PAA-TPE_*x*_)

*F*_max_: Maximum fluorescence intensity (for PAA-TPE_*x*_) or quantum yield (for g-PAA-TPE_*x*_)

*F*_min_: Minimum fluorescence intensity (for PAA-TPE_*x*_) or quantum yield (for g-PAA-TPE_*x*_)

*K*_d_: Apparent dissociation constant

*n*: Apparent Hill coefficient

### Evaluation of the swelling ratio of g-PAA-TPE_*x*_

For swelling the gel, a sliced sample was immersed in a HEPES buffer solution (70 mM, pH = 7.4) at 25 °C for 30 minutes. Swelling ratios were evaluated from the following equation (2):


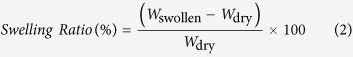


*W*_dry_: The weight of dried g-PAA-TPE_*x*_

*W*_swollen_: The weight of swollen g-PAA-TPE_*x*_

### Animal experiments

All experimental protocols were approved by the Animal Care Committee of Nara Medical University according to the NIH (USA) guidelines and the Guidelines for Proper Conduct of Animal Experiments published by the Science Council of Japan. Experimental details are described in Supplementary Information.

## Additional Information

**How to cite this article**: Ishiwari, F. *et al.* Bioinspired design of a polymer gel sensor for the realization of extracellular Ca^2+^ imaging. *Sci. Rep.* **6**, 24275; doi: 10.1038/srep24275 (2016).

## Supplementary Material

Supplementary Information

Supplementary Movie S1

Supplementary Movie S2

## Figures and Tables

**Table 1:** Structural parameters and Ca^2+^-sensing properties of PAA-TPE_*x*_ and g-PAA-TPE_*x*_.

Entry	PAA-TPE_*x*_ or g-PAA-TPE_*x*_	Molar Ratio of TPE (*x*)*	Molar Ratio of PAA (*y*)*	Molar Ratio of Crosslinker (*z*)*	*M*_n_ (kDa)^†^	PDI^†^	Swelling Ratio (%)^‡^	Apparent *K*_d_ for Ca^2+^ (mM)^§^	Dynamic Range^#^
1	PAA-TPE_0.01_	0.01	0.99	–	20	1.95	–	2.8	24
2	PAA-TPE_0.02_	0.02	0.98	–	24	1.98	–	1.8	69
3	PAA-TPE_0.03_	0.03	0.97	–	26	2.01	–	0.77	33
4	PAA-TPE_0.04_	0.04	0.96	–	17	1.99	–	0.65	12
5	PAA-TPE_0.05_	0.05	0.95	–	25	1.59	–	0.43	5.5
6	g-PAA-TPE_0.01_	0.01	0.96	0.03	–	–	4,800	13	7.1
7	g-PAA-TPE_0.02_	0.02	0.95	0.03	–	–	2,960	5.7	12
8	g-PAA-TPE_0.03_	0.03	0.94	0.03	–	–	1,530	3.5	8.3
9	g-PAA-TPE_0.04_	0.04	0.93	0.03	–	–	660	2.0	5.9
10	g-PAA-TPE_0.05_	0.05	0.92	0.03	–	–	580	2.0	4.4

^*^Determined by ^1^H NMR spectroscopy for PAA-TPE_*x*_ (Supplementary Fig. S20), and defined as feed ratios for g-PAA-TPE_*x*_. ^†^Estimated by GPC analysis of the corresponding precursor polymers (*t*-Bu-PAA-TPE_*x*_) with *t*-butyl groups (see Methods for details). ^‡^Determined after immersion in a buffer solution for 30 minutes at 25 °C (see Methods for details). ^§^Determined by fitting the Ca^2+^ titration curves (Fig. 3b,d) using the Hill equation (see Methods for details). ^#^Defined as the ratio of the maximum to the minimum fluorescence intensity.
