# Supplementary material for: Bioinspired design of a polymer gel sensor for the realization of extracellular Ca2+ imaging
Source: Sci Rep. 2016 Apr 12;6:24275. doi: 10.1038/srep24275 (PMC4828671; doi:10.1038/srep24275)
Supplement: Supplementary Information [file srep24275-s1.pdf]

SUPPLEMENTARY INFORMATION

Bioinspired design of a polymer gel sensor for the realization of extracellular Ca^{2+} imaging

Fumitaka Ishiwari, Hanako Hasebe, Satoko Matsumura, Fatin Hajjaj, Noriko Horii-Hayashi, Mayumi Nishi, Takao Someya and Takanori Fukushima*

*To whom correspondence should be addressed.
E-mail: fukushima@res.titech.ac.jp (T.F.)

Table of Contents

1. General........S2
2. Preparation Method of Mouse Brain Slice........S2
3. Ca^{2+} Imaging by Stamp Experiment........S3
4. *In situ* Ca^{2+} Imaging with a Fluorescence-Inverted Microscope........S3
5. Supplementary Figures (Supplementary Figs. S1–S16)........S4
6. Analytical Data (Supplementary Figs. S17–S21)........S20
7. Legends of Movies........S23

1. General

Unless otherwise noted, all the experiments were conducted at 25 °C. Analytical GPC was performed at 40 °C regulated by column oven (CO-2065*Plus*) on a JASCO HSS-1500 system equipped with a refraction index (RI) detector (RI-2031*Plus*) and a multiwavelength detector (MD-2010*Plus*), using dimethylformamide (DMF) containing LiBr (0.01 M) as an eluent at a flow rate of 0.80 mL/min on a column (TSKgel α-3000, TOSOH). The molecular weight calibration curve was obtained by using standard polystyrenes (TSKstandard polystyrene, TOSOH). Nuclear magnetic resonance (NMR) spectra were recorded at 25 °C on a Bruker model AVANCE-400 spectrometer (400.0 MHz for ^{1}H and 100.6 MHz for ^{13}C), where chemical shifts (δ) were determined with respect to residual non-deuterated solvent for ^{1}H ($CDCl_3$: ^{1}H(δ) = 7.26 ppm, CD_3OD ^{1}H(δ) = 3.31 ppm), residual solvent for ^{13}C ($CDCl_3$: ^{13}C(δ) = 78.0 ppm). The absolute values of the coupling constants are given in Hertz (Hz), regardless of their signs. Multiplicities are abbreviated as singlet (s), doublet (d), triplet (t), quartet (q), multiplet (m) and broad (br). Infrared (IR) spectra were recorded at 25 °C on a JASCO model FT/IR-660*Plus* Fourier transform infrared spectrometer. High-resolution fast atom bombardment mass spectrometry (FAB-MS) measurements were performed on a JEOL model JMS-700MStation mass spectrometer. Electronic absorption spectra were recorded using a quartz cell on a JASCO model V-670 UV/VIS spectrophotometer. Fluorescence spectra were recorded using a quartz cell on a JASCO model FP-6500 spectrophotometer. Absolute fluorescence quantum yields were measured using a quartz cell at 25 °C on a Hamamatsu Photonics model C9920-03. Unless otherwise noted, all the fluorescence properties and absolute fluorescence quantum yields were measured by excitation at 307 nm (λ_{ex} = 307 nm). Dynamic light scattering experiments were performed at 25 °C on a Wyatt technology model DynaPro® NanoStar™ using a 45 μL quartz cuvette, where the data were recorded and analyzed using a DYNAMICS® software system. Slicing operations of g-PAA-TPE$_x$ were carried out using a Dosaka Em model ZERO-1® microslicer® or a Leica model Ultracut UCT ultramicrotome. Swollen g-PAA-TPE$_x$ was sliced into *ca*. 200 μm-thick sections by a microslicer. Grinding operations of g-PAA-TPE$_x$ were performed on a Fritsch model P-7 Classic-Line ball mill using 15 mm, 5 mm and 1 mm zirconia balls (ZrO_2: 96.4%, MgO: 3.2%, Other: 0.4%). Fluorescence images were taken by a Canon model EOS Kiss X7i camera under UV light irradiation using an AS ONE model SUV-4 254 nm handy UV lamp and analyzed by ImageJ software (http://imagej.nih.gov/ij/). Fluorescence monitoring using a flow channel was performed on a Nippon Sheet Glass model FLE1100 optical fiber fluorescence detector, where solutions were flowed by a KD scientific model KDS 100 syringe pump.

2. Preparation method of mouse brain slice

All experimental protocols were approved by the Animal Care Committee of Nara Medical University according to the NIH (USA) guidelines and the Guidelines for Proper Conduct of Animal Experiments published by the Science Council of Japan. Male C57BL/6N mice were

purchased from Japan CLEA Inc. (Tokyo, Japan) and housed with free access to food and water under standard laboratory conditions (23 °C, 55% humidity, and a 12-hour light–dark cycle: lights on at 8:00 a.m./lights off at 8:00 p.m.). Mice at 11 weeks old were anesthetized with sevoflurane and euthanized by decapitation. Dissected brains immersed in cold phosphate-burred saline (PBS, pH = 7.5) were sliced with 200 μm-thick using a microslicer (Dosaka, Kyoto, Japan). Brain slices were mounted on coverslips and dried up.

3. Ca^{2+} imaging by stamp experiment

A mouse brain slice was placed on a sheet of g-PAA-$TPE_{0.02}$ for 1 minute through a dialysis membrane (Spectrum® Laboratories Inc., Biotech Regenerated Cellulose Membrane, Molecular Weight Cut Off 3,500–5,000). After removal of the slice and dialysis membrane, fluorescent signal was observed under UV irradiation (Supplementary Figs. S13 and S14). As a control, the same experiment was performed using a mouse brain slice pre-immersed in an EDTA solution to chelate Ca^{2+} in tissues.

4. *In situ* Ca^{2+} imaging with a fluorescence-inverted microscope

A set of g-PAA-$TPE_{0.02}$ slice, dialysis membrane and mouse brain slice sandwiched between two coverslips was observed using a fluorescence-inverted microscope equipped with the WU filter cube (IX81, Olympus, Tokyo, Japan). When performing an experiment for Ca^{2+} chelation, PBS containing 10 mM EDTA was dropped on mouse brain slices dried up on coverslips and they were kept for 30 minutes in a humidified chamber. The slices were washed with PBS, and dried up again. Methods for subsequent microscopic observation were the same as above. Capturing of images was performed under the same microscopic conditions in exposure time and binning.

5. Supplementary Figures

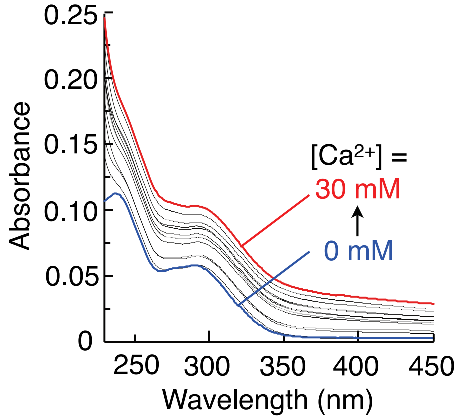

Supplementary Fig. S1 | Absorption spectral change of PAA-$TPE_{0.02}$ (10 mg/L) in a HEPES buffer solution (70 mM, pH = 7.4) at 25 °C upon addition of $CaCl_2$ (blue: 0 mM → red: 30 mM).

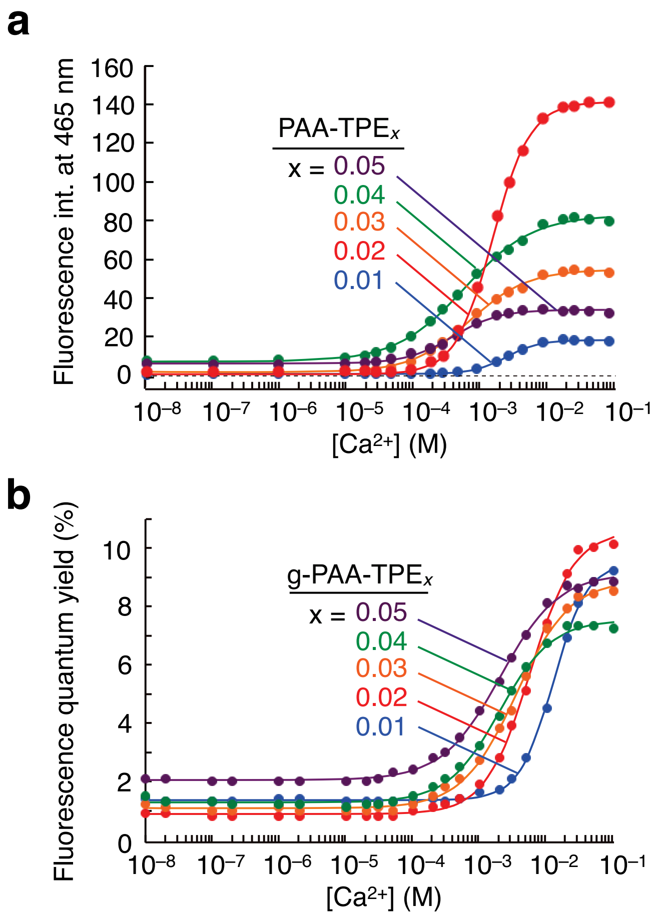

Supplementary Fig. S2 | a,**b**, Ca^{2+} titration curves of (**a**) PAA-TPE$_x$ (10 mg/L) in a HEPES buffer solution (70 mM, pH = 7.4) and (**b**) g-PAA-TPE$_x$ (5 mg) in a HEPES buffer solution (70 mM, 5 mL, pH = 7.4).

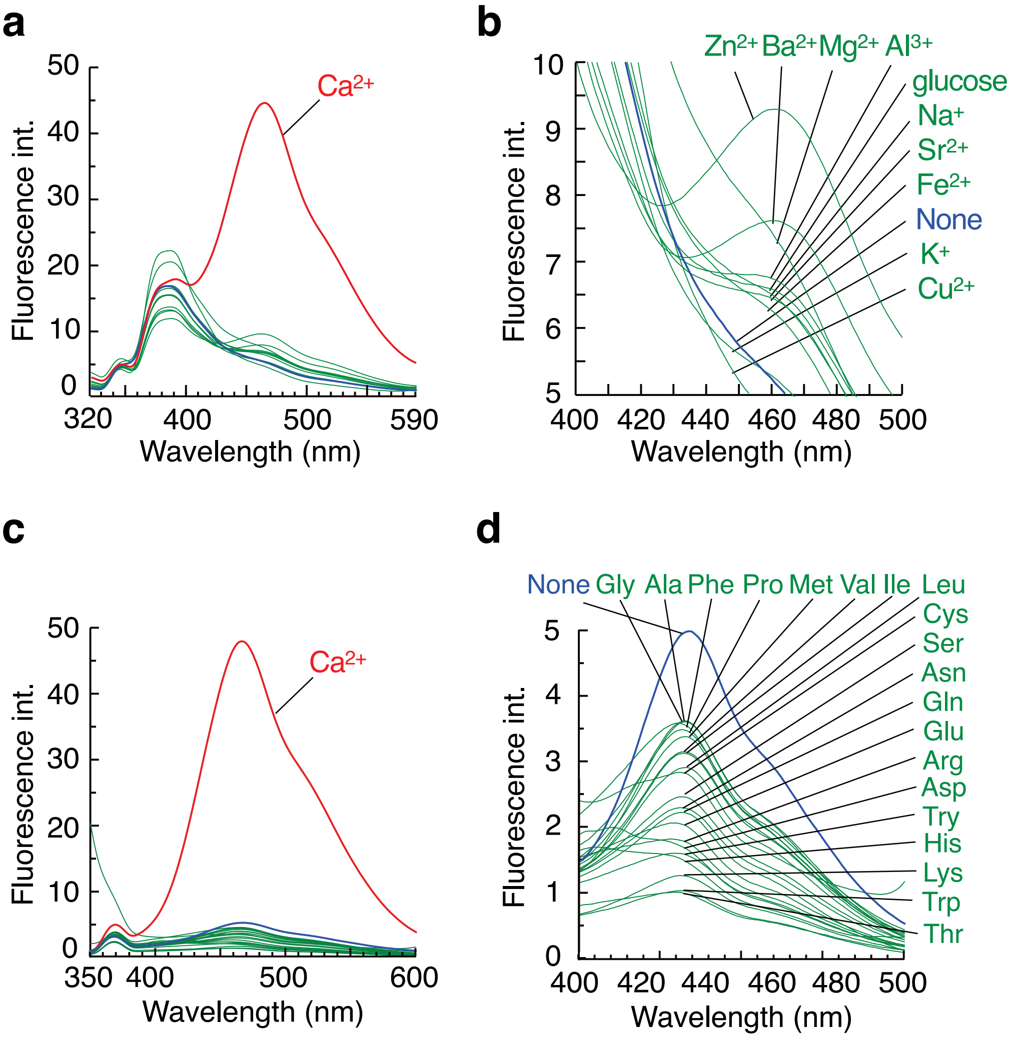

Supplementary Fig. S3 | a,b, Fluorescence spectra (λ_{ex} = 307 nm) of PAA-$TPE_{0.02}$ (10 mg/L) in a HEPES buffer solution (70 mM, pH = 7.4) at 25 °C in the presence of various metal chlorides and glucose (**a**), and their magnifications (**b**). [$CaCl_2$] = [$MgCl_2$] = 2 mM, [NaCl] = 145 mM, [KCl] = 5 mM, [$FeCl_2$] = [$CuCl_2$] = [$ZnCl_2$] = [$AlCl_3$] = [$SrCl_2$] = [$BaCl_2$] = 50 μM and [glucose] = 14 mM. **c**,**d**, Fluorescence spectra (λ_{ex} = 325 nm) of PAA-$TPE_{0.02}$ (10 mg/L) in a HEPES buffer solution (70 mM, pH = 7.4) at 25 °C in the presence of various amino acids (5 mM) (**c**), and their magnifications (**d**). Ala = alanine, Arg = arginine, Asn = asparagine, Asp = aspartic acid, Cys = cysteine, Glu = glutamic acid, Gln = glutamine, Gly = glycine, His = Histidine, Ile = isoleucine, Leu = leucine, Lys = lysine, Met = methionine, Phe = phenylalanine, Pro = proline, Ser = serine, Thr = threonine, Trp = tryptophan, Tyr = tyrosine and Val = valine.

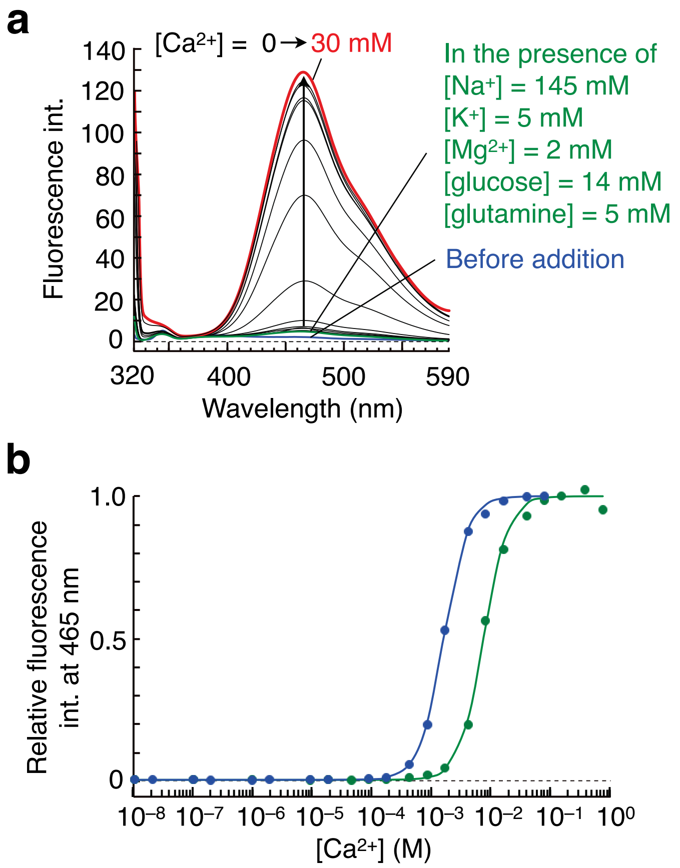

Supplementary Fig. S4 | a, Fluorescence spectral changes of PAA-TPE$_{0.02}$ (10 mg/L) in a HEPES buffer solution (70 mM, pH = 7.4) at 25 °C before (blue) and after (green) addition of a mixture of NaCl (145 mM), KCl (5 mM), $MgCl_2$ (2 mM), glucose (14 mM) and glutamine (5 mM), and after further addition of $CaCl_2$ (black: 0 mM → red: 30 mM). **b**, Ca^{2+} titration curves of PAA-TPE$_{0.02}$ in the absence (blue) and presence (green) of a mixture of NaCl (145 mM), KCl (5 mM), $MgCl_2$ (2 mM), glucose (14 mM) and glutamine (5 mM). The relative fluorescence intensity is defined as $(F–F_{min})/(F_{max}–F_{min})$, where F, F_{max} and F_{min} represent observed, maximum and minimum fluorescence intensities, respectively.

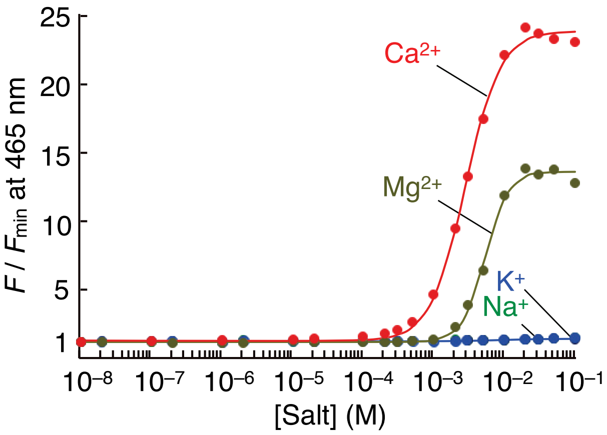

Supplementary Fig. S5 | Titration curves of PAA-$TPE_{0.01}$ (10 mg/L) with NaCl, KCl, $MgCl_2$ and $CaCl_2$ in a HEPES buffer solution (70 mM, pH = 7.4) at 25 °C. F and F_{min} represent the observed and minimum fluorescence intensities at 465 nm, respectively. Apparent K_d values for Ca^{2+} and Mg^{2+} were determined to be 2.8 and 5.8 mM, respectively, and dynamic ranges for Ca^{2+} and Mg^{2+} were 24 and 13, respectively.

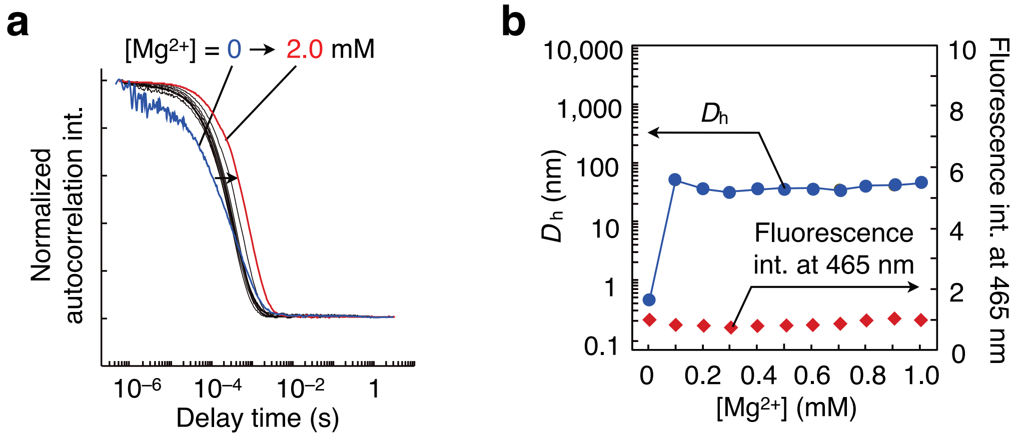

Supplementary Fig. S6 | Mg^{2+}-triggered aggregation behavior of PAA-TPE$_{0.02}$. **a**, Changes in the autocorrelation functions of PAA-TPE$_{0.02}$ (10 mg/L) at 25 °C in a water/methanol mixture (1/1 v/v) containing various concentrations of $MgCl_2$ obtained by dynamic light scattering measurements. **b**, Mg^{2+}-concentration dependence of the logarithms of hydrodynamic diameter (D_h) and fluorescence intensity of PAA-TPE$_{0.02}$ at 465 nm. We confirmed the difference in the aggregation behavior of PAA-TPE$_{0.02}$ in the presence of Mg^{2+} and Ca^{2+} (Fig. 4a,b). It has been reported that, unlike in the case of Ca^{2+}[40–44], the carboxyl group of PAA tends to coordinate to Mg^{2+} with a geometry of [CO_2^---Mg^{2+}] + CO_2^- rather than [CO_2^---Mg^{2+}--^-O_2C][41]. Because of this preference in the coordination pattern, PAA-TPE$_{0.02}$ may have a larger apparent K_d value for Mg^{2+} (Supplementary Fig. S5) than for Ca^{2+}, and thus may be able to clearly distinguish Ca^{2+} from Mg^{2+}.

a

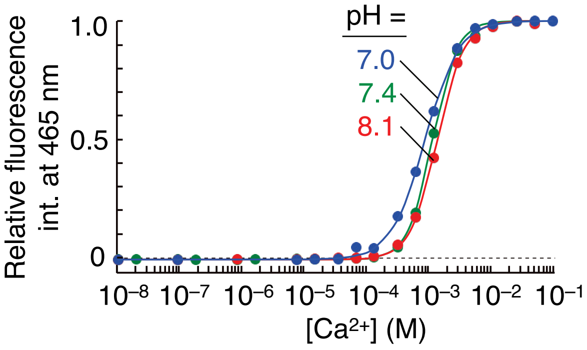

b

pH	Apparent K_d for Ca^{2+} (mM)	Dynamic Range	Hill Coefficient
7.0	1.4	53	1.5
7.4	1.9	67	2.1
8.1	2.3	49	1.9

Supplementary Fig. S7 | a,**b**, Ca^{2+}-titration experiments of PAA-$TPE_{0.02}$ under various pH conditions. Ca^{2+}-titration curves (**a**) and Ca^{2+}-sensing properties (**b**) of PAA-$TPE_{0.02}$ (10 mg/L) in a HEPES buffer solution (70 mM) at 25 °C under various pH conditions.

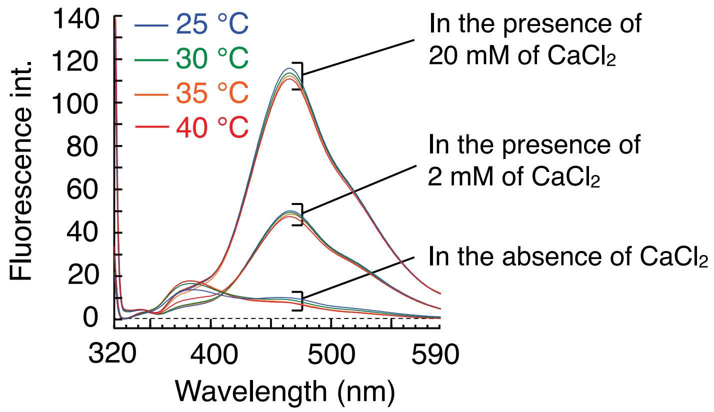

Supplementary Fig. S8 | Fluorescence spectra of PAA-TPE$_{0.02}$ (10 mg/L) in a HEPES buffer solution (70 mM, pH = 7.4) at 25 (blue), 30 (green), 35 (orange) and 40°C (red) in the absence and presence of 2 and 20 mM of $CaCl_2$.

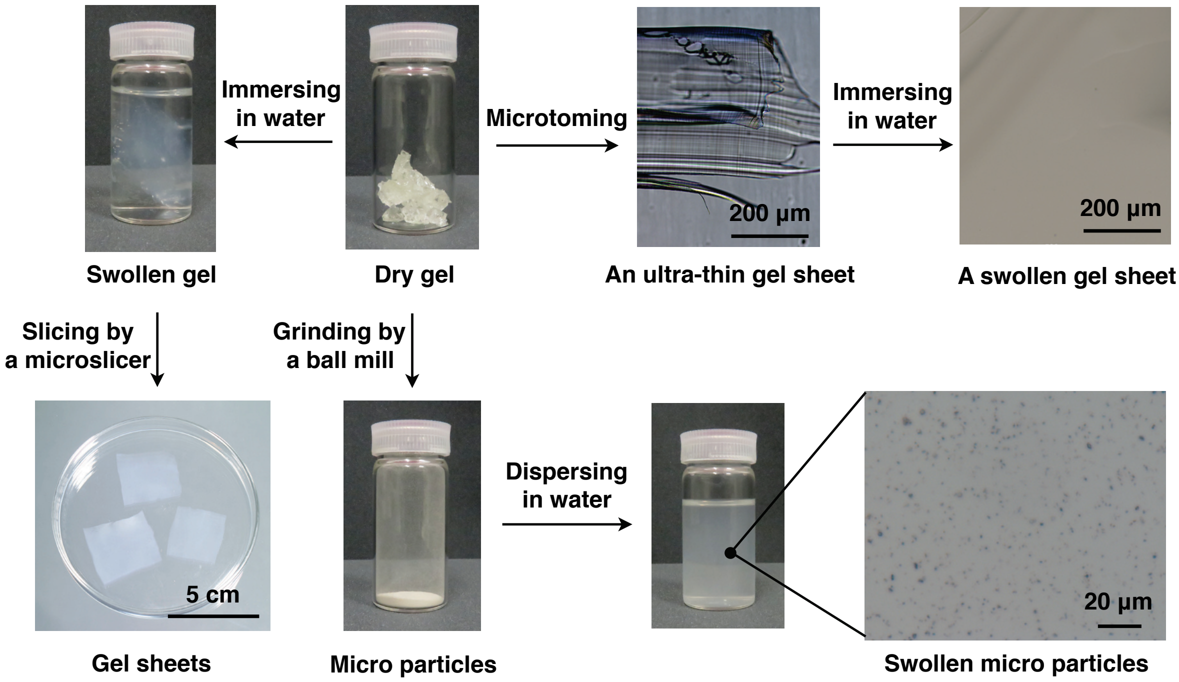

Supplementary Fig. S9 | Processability of g-PAA-TPE$_{0.02}$. The gel can be readily processed into various shapes and size regimes.

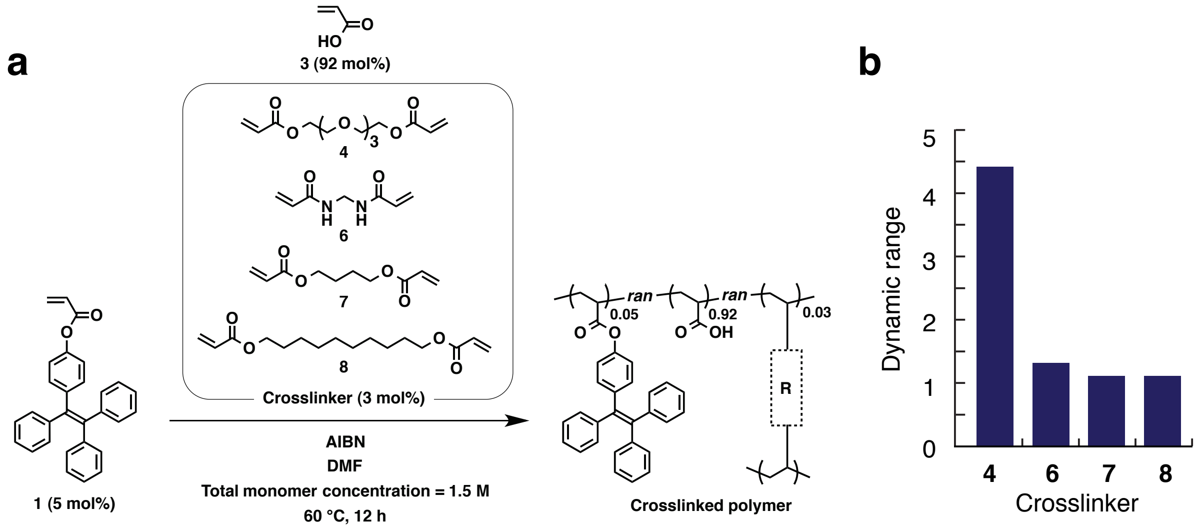

Supplementary Fig. S10 | Effect of crosslinker on the dynamic range of TPE-appended crosslinked polymers. **a**, Synthetic scheme for crosslinked polymers using various crosslinkers; tetraethyleneglycol diacrylate (**4**), *N*,*N*'-methylenebisacrylamide (**6**), 1,4-butanediol diacrylate (**7**) and 1,10-decanediol diacrylate (**8**). **b**, Dynamic ranges observed for the resultant crosslinked polymers upon addition of Ca^{2+} (10 mM). When diacrylic compounds **6**–**8** were used as crosslinkers, the resultant gels scarcely showed an increase in fluorescence upon the addition of Ca^{2+}, where the values of the dynamic range were as low as 1.

a

Entry	Total monomer concentration (M)	Feed ratio of **1** (mol%)	Feed ratio of acrylic acid **3** (mol%)	Feed ratio of crosslinker **4** (mol%)	Swelling ratio (%)	Dynamic range
1	4.0	2.0	95.0	3.0	590	2.3
2	4.0	2.0	96.5	1.5	620	2.5
3	4.0	2.0	97.0	1.0	670	2.8
4	4.0	2.0	97.5	0.5	750	3.2
5	4.0	2.0	97.9	0.1	No gelation	–
6	2.0	2.0	95.0	3.0	1,510	7.4
7	2.0	2.0	96.5	1.5	1,820	8.1
8	2.0	2.0	97.0	1.0	No gelation	–
9	1.5	2.0	95.0	3.0	2,960	12
10	1.5	2.0	96.5	1.5	No gelation	–
11	1.0	2.0	95.0	3.0	No gelation	–

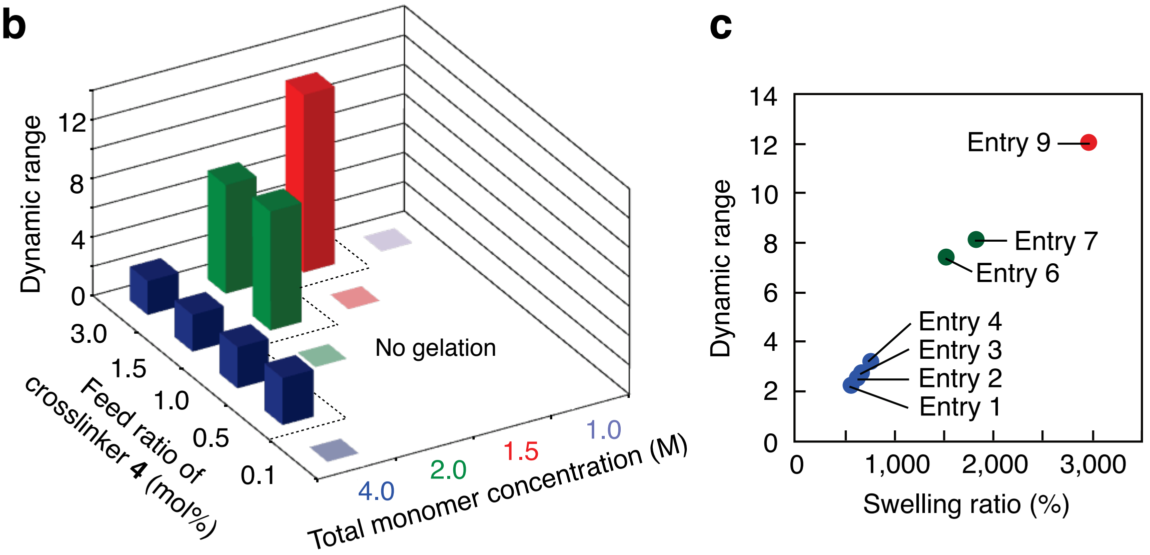

Supplementary Fig. S11 | Preparation conditions of g-PAA-TPE$_{0.02}$ for optimizing the swelling ratio and dynamic range for Ca^{2+} sensing. **a**, Summary of the effect of the total monomer concentration and the feed ratios of compounds **1**, **3** and **4** on the swelling ratio and dynamic range of g-PAA-TPE$_{0.02}$. **b**, Block graph expression of the relationship among dynamic range, the feed ratio of crosslinker **4** and the total monomer concentration. The values of dynamic ranges were evaluated upon addition of Ca^{2+} (30 mM). **c**, Plot of dynamic range versus swelling ratio.

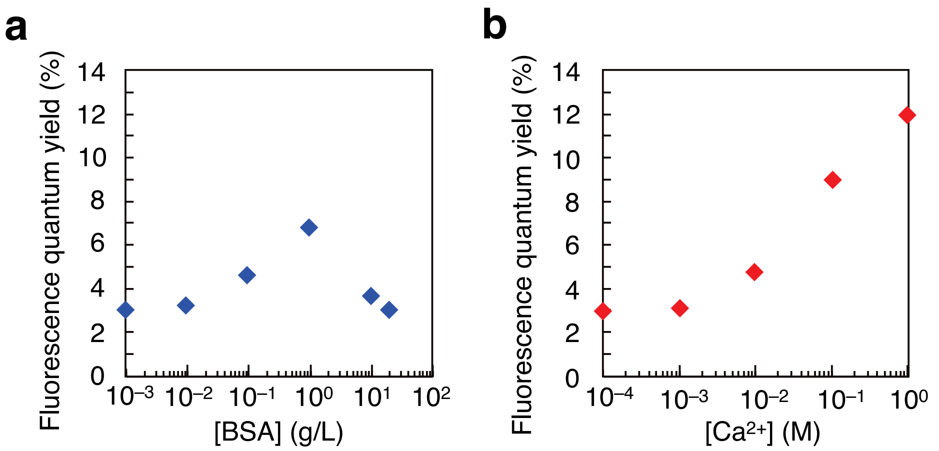

Supplementary Fig. S12 | a, BSA (bovine serum albumin) titration curve of g-PAA-TPE$_{0.02}$ (5 mg) in a HEPES buffer solution (70 mM, 5 mL, pH = 7.4) at 25 °C. **b**, Ca^{2+} titration curves of g-PAA-TPE$_{0.02}$ (5 mg) in a HEPES buffer solution (70 mM, 5 mL, pH = 7.4) at 25 °C in the presence of BSA (20 g/L). Fluorescence quantum yields were measured by excitation at 325 nm.

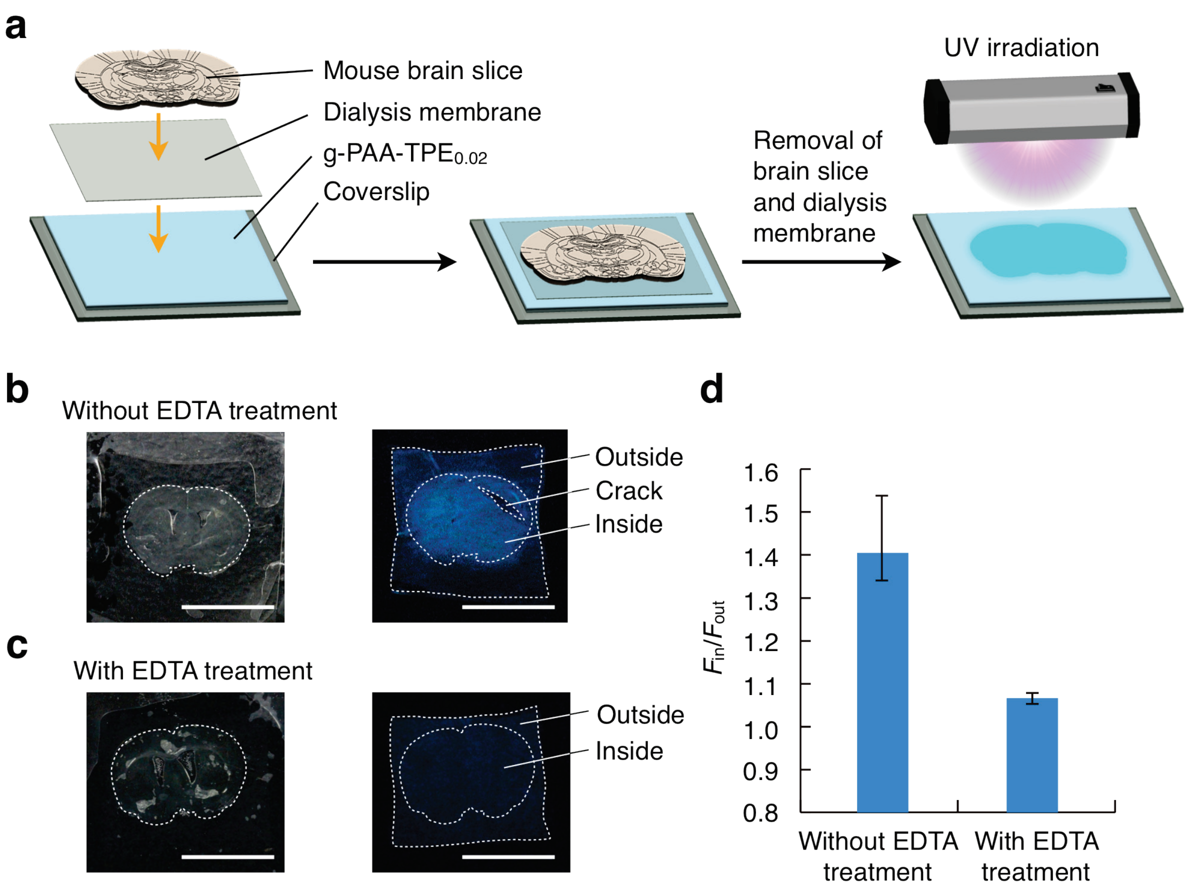

Supplementary Fig. S13 | a, Schematic illustration of fluorescence Ca^{2+} imaging of a mouse brain slice using g-PAA-$TPE_{0.02}$. **b**,**c**, The left pictures show gel sheets of g-PAA-$TPE_{0.02}$ overlaid with mouse brain slices without (**b**) or with (**c**) EDTA treatment to chelate Ca^{2+} in the brain slices; the right pictures indicate fluorescent images obtained after removal of the slices followed by UV irradiation. The white dotted lines indicate the outlines of the brain slices and gel sheets. **d**, Fluorescence increasing ratios of inside and outside areas in each gel sheet (F_{in}/F_{out}; see also the right pictures in **b** and **c**). The values are the average of four experiments. Cracked areas of the gel, if any, were excluded from the evaluation. Scale bars, 1.0 cm.

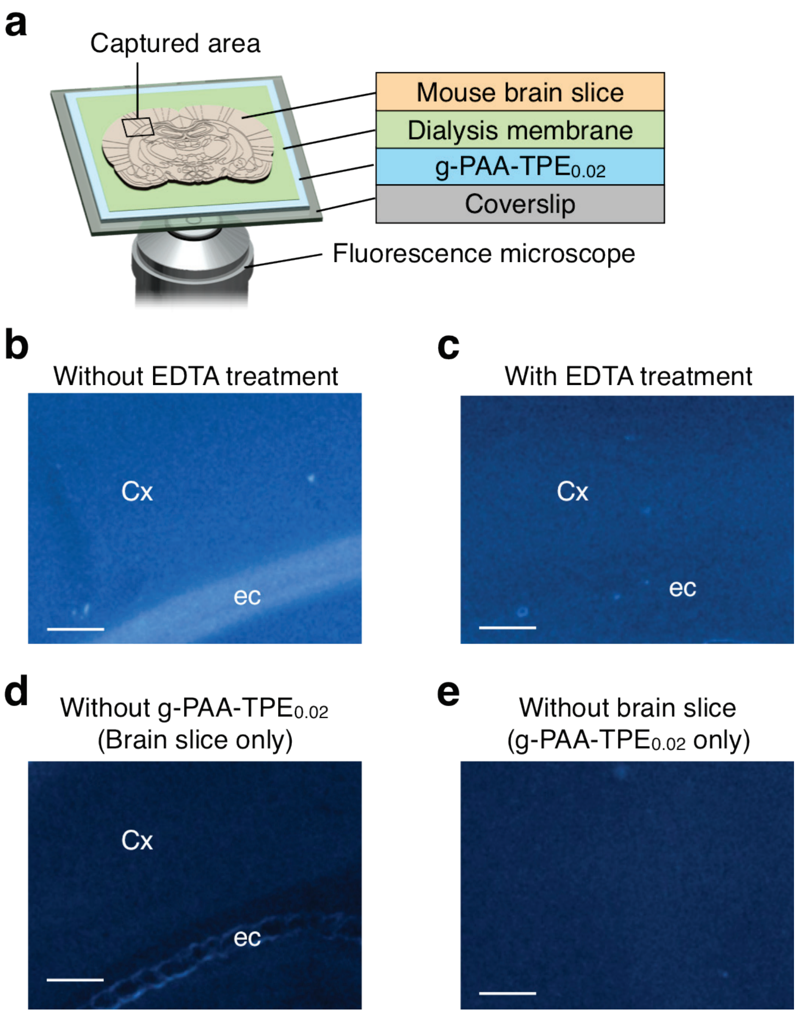

Supplementary Fig. S14 | a–e, *In situ* Ca^{2+} imaging on mouse brain slices with a fluorescence-inverted microscope. Schematic illustration for the preparation of imaging samples (**a**). A slice of g-PAA-$TPE_{0.02}$ mounted on a coverslip was overlaid with a dialysis membrane, and then mouse brain slices were put on the dialysis membrane just before microscopic observation (**b**–**e**). All fluorescence images were captured under the same microscopic conditions including exposure time and binning. Fluorescent Ca^{2+} signal (blue) was observed in the combination of a non-pretreated brain slice and g-PAA-$TPE_{0.02}$ (**b**), while it was not observed in the combination of an EDTA-pretreated brain slice and g-PAA-$TPE_{0.02}$ (**c**), a brain slice only (**d**) and g-PAA-$TPA_{0.02}$ only (**e**). Cx, cerebral cortex; ec, external capsule. Scale bars, 100 μm.

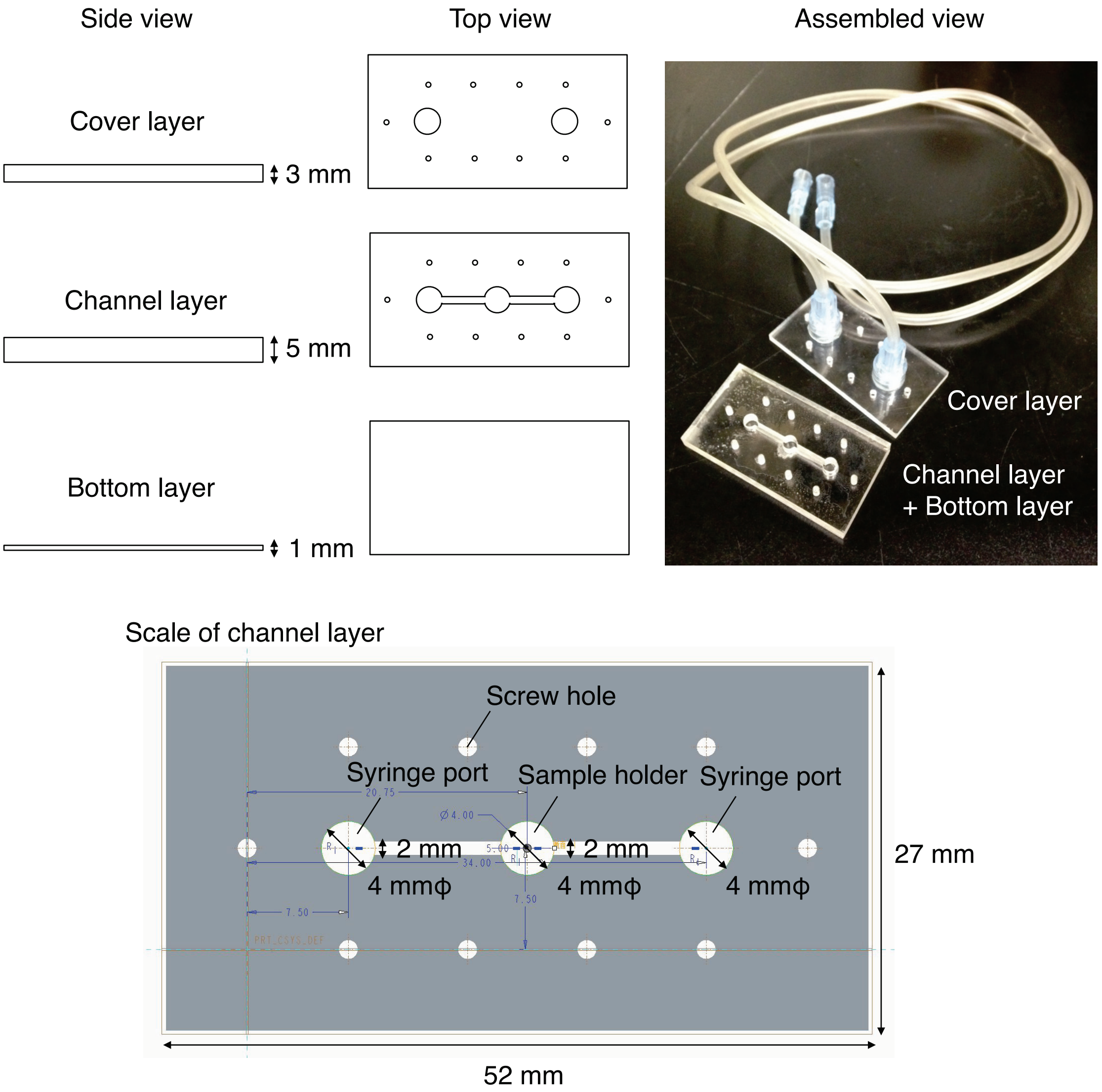

Supplementary Fig. S15 | Schematic illustrations and a photograph of the flow channel used for continuous monitoring (Fig. 5h–j) of the change in Ca^{2+} concentration with g-PAA-$TPE_{0.02}$.

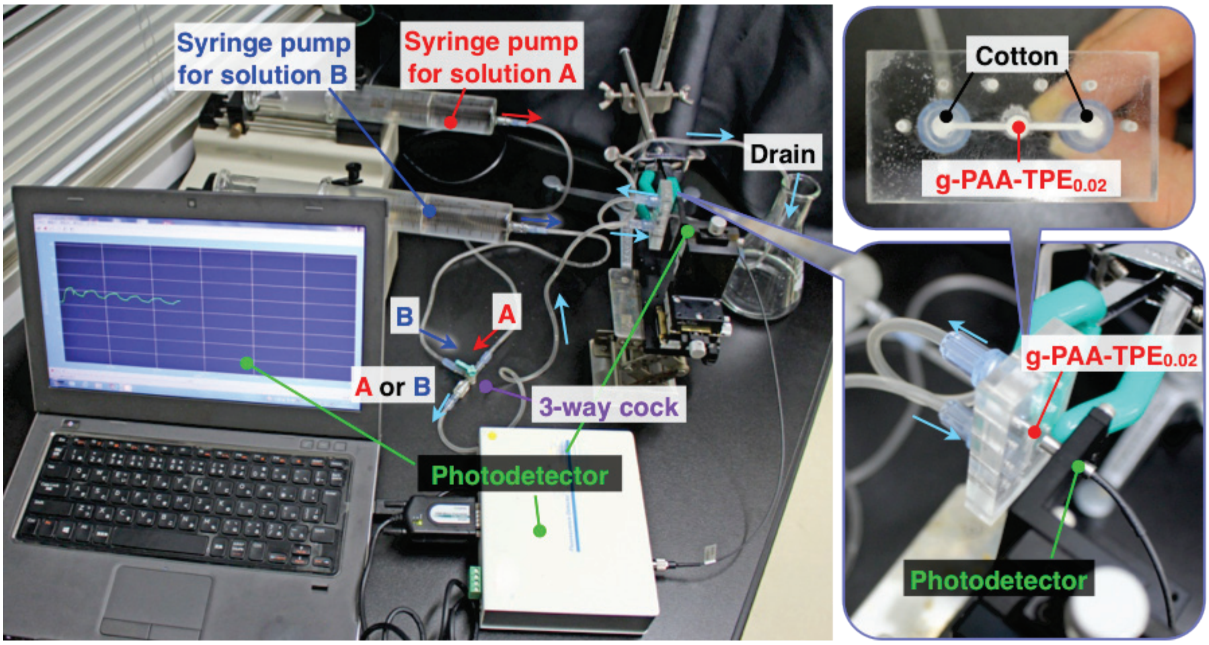

Supplementary Fig. S16 | Photographs of the experimental setup for continuous monitoring (Fig. 5h–j) of the change in Ca^{2+} concentration with g-PAA-TPE$_{0.02}$.

6. Analytical Data

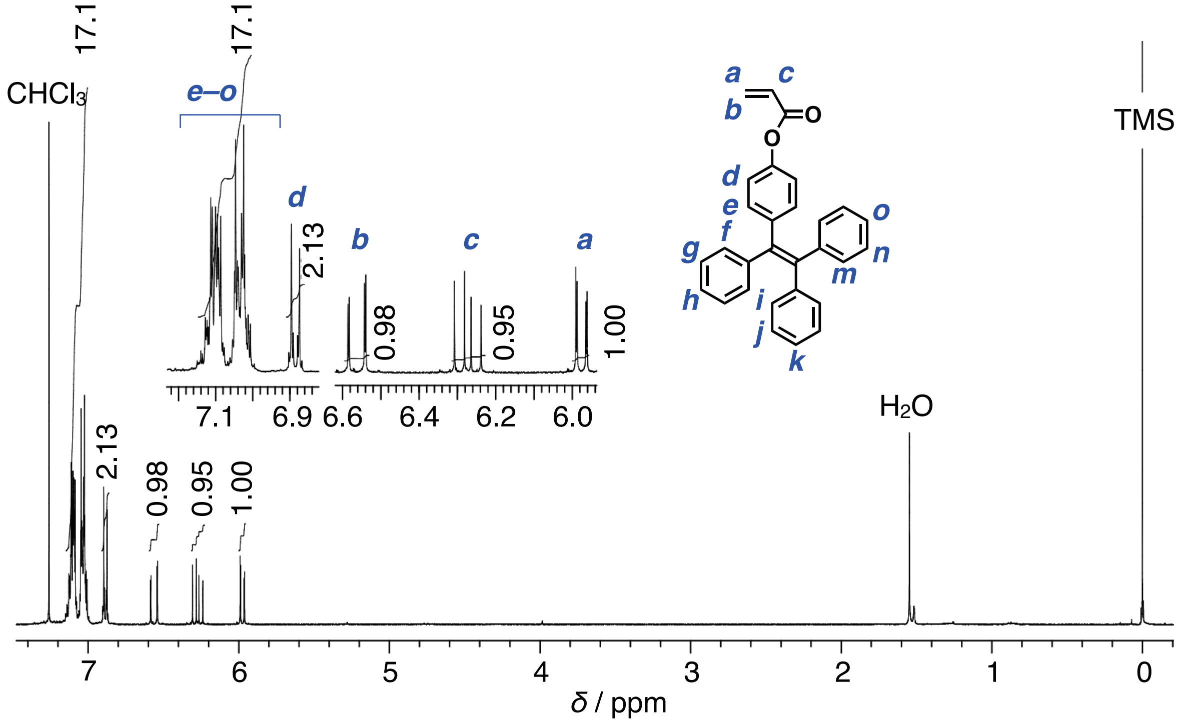

Supplementary Fig. S17 | ^{1}H NMR spectrum (400 MHz) of **1** in $CDCl_3$ at 25 °C.

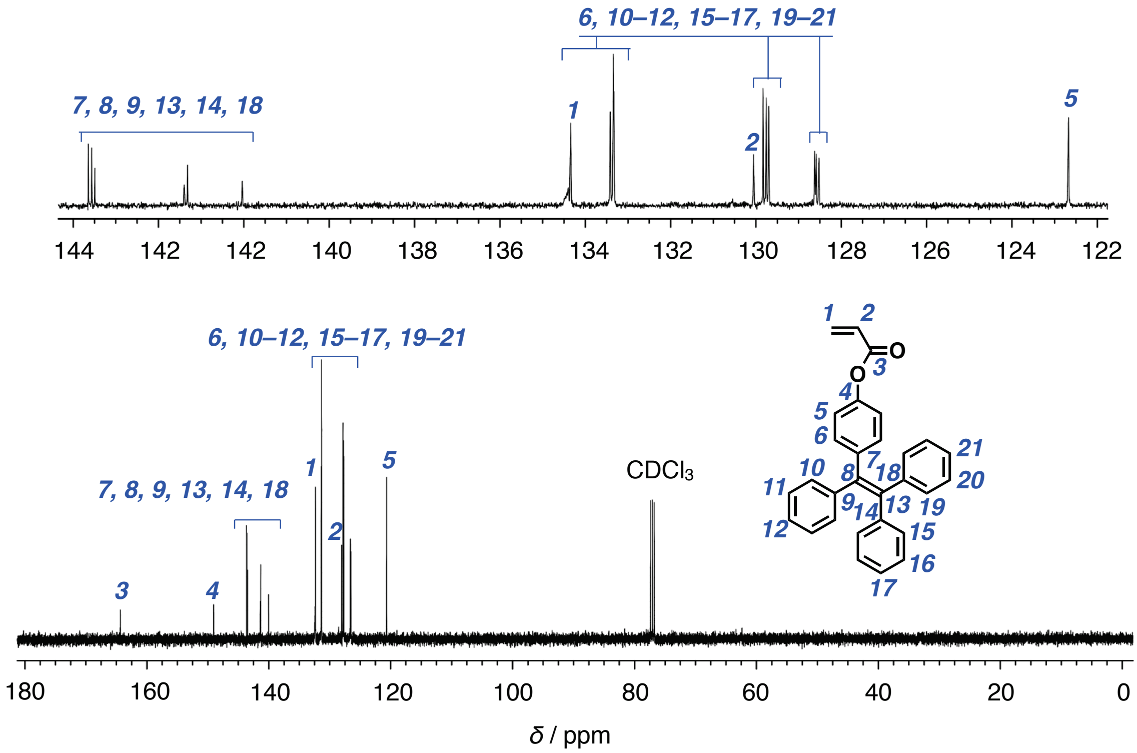

Supplementary Fig. S18 | ^{13}C NMR spectrum (100 MHz) of **1** in $CDCl_3$ at 25 °C.

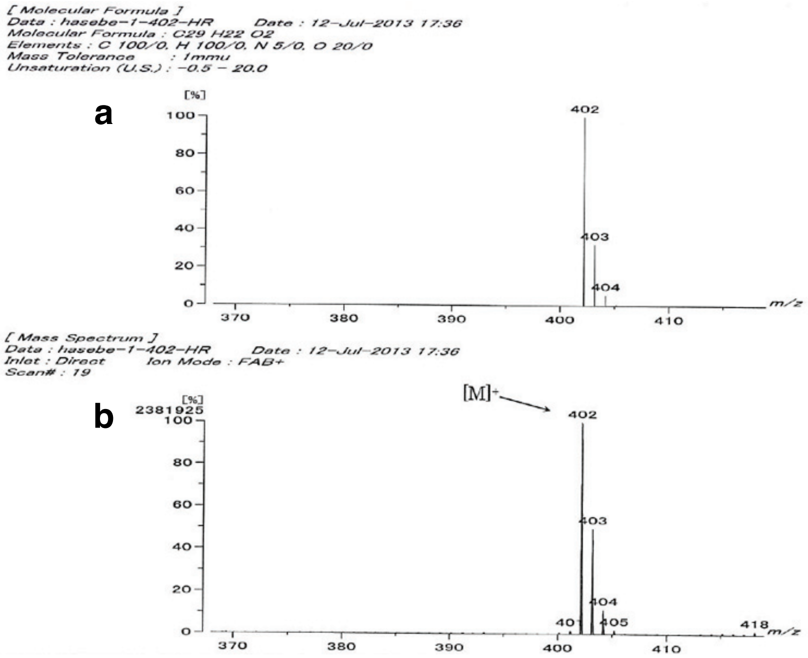

Supplementary Fig. S19 | a,**b**, Simulated (**a**) and observed (**b**) high-resolution FAB-MS spectra of **1**.

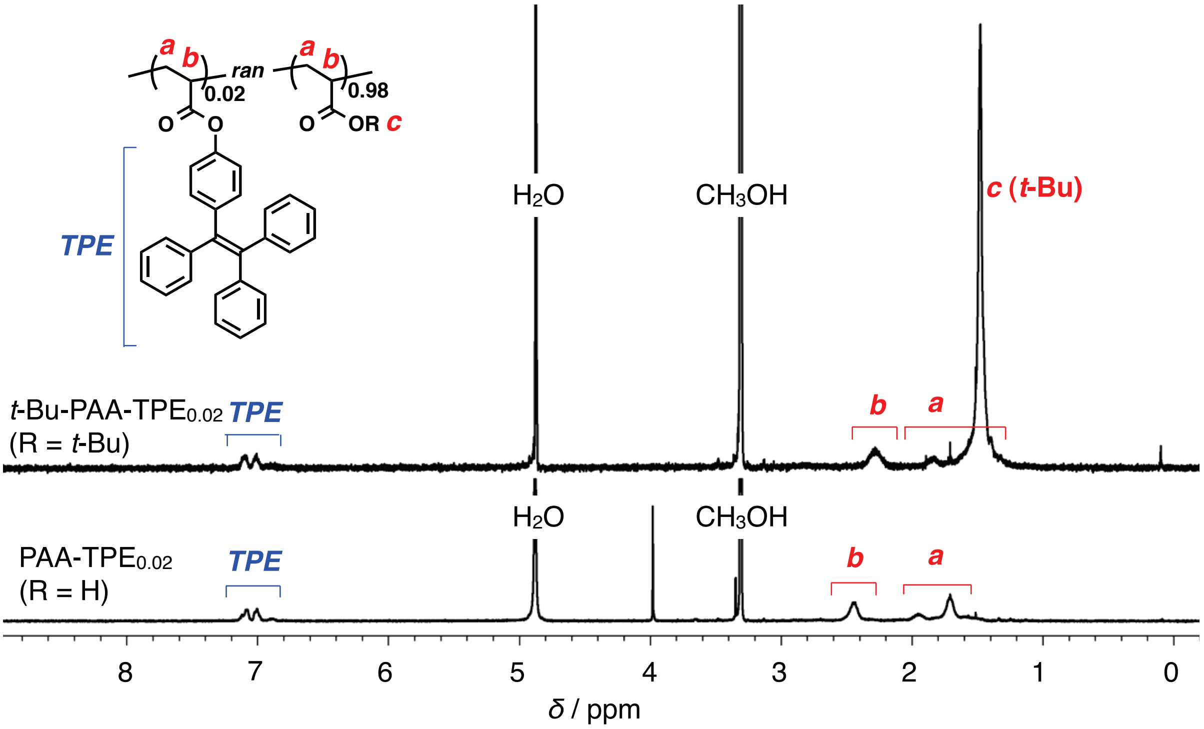

Supplementary Fig. S20 | Typical ^{1}H NMR spectra of polyacrylic acid derivatives (PAA-TPE$_x$). ^{1}H NMR spectra (400 MHz) of PAA-TPE$_{0.02}$ and *t*-Bu-PAA-TPE$_{0.02}$ in CD_3OD at 25 °C.

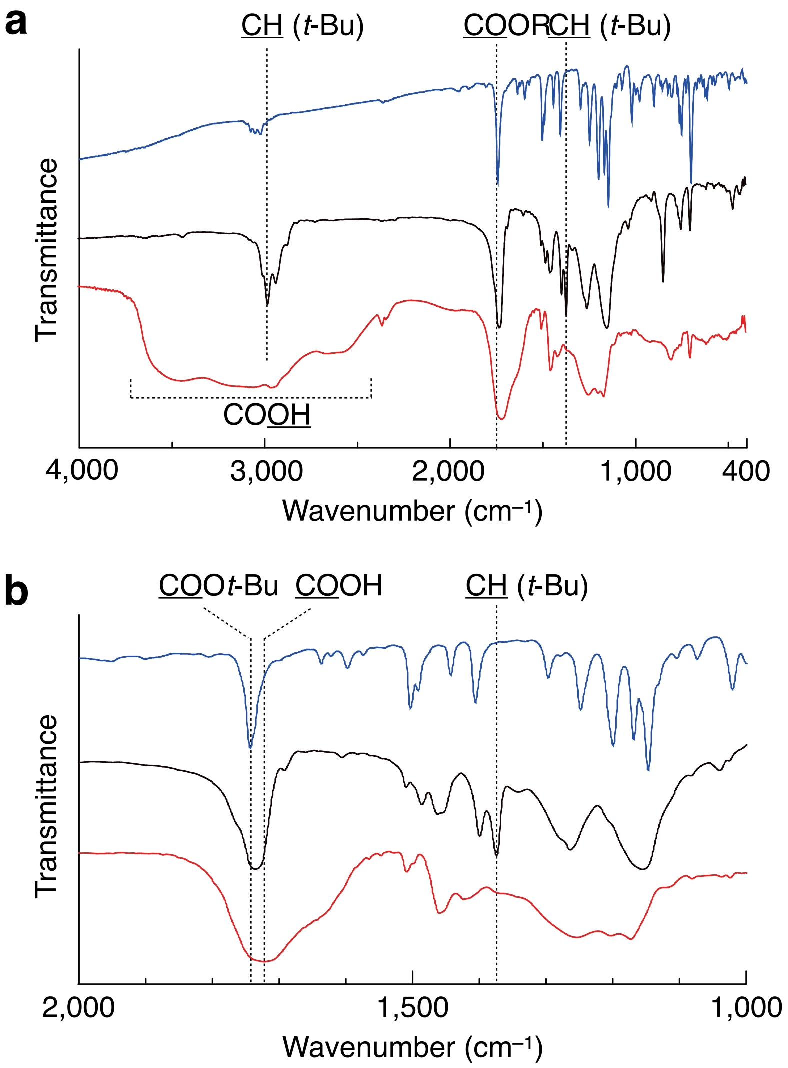

Supplementary Fig. S21 | a,**b**, FT-IR spectra (KBr) of **1** (blue), *t*-Bu-PAA-TPE$_{0.02}$ (black) and PAA-TPE$_{0.02}$ (red) as typical examples of polyacrylic acid derivatives (**a**), and their magnifications (**b**).

7. Legends of Movies

Supplementary Movie S1 | Real-time image of the fluorescence emission of g-PAA-$TPE_{0.02}$ upon contacting with a droplet of a HEPES buffer solution (70 mM, pH = 7.4) containing $CaCl_2$ (30 mM).

Supplementary Movie S2 | Spatiotemporal imaging of the diffusion of Ca^{2+}. Five pieces of g-PAA-$TPE_{0.02}$ (ca. 5 mm × 5 mm × 200 μm) were placed in a Petri dish at an interval of 1 cm and immersed in a HEPES buffer solution (70 mM, 10 mL, pH = 7.4), and then a concentrated $CaCl_2$ solution (200 μL, 100 mM) was dropped at the right side of the rightmost gel. With diffusion of Ca^{2+}, the aligned gel samples started to emit fluorescence sequentially from right to left sides.
